# Near-term, geospatial opportunity for biomass carbon storage to address the wildfire and climate crises

**DOI:** 10.1126/sciadv.aee6185

**Published:** 2026-08-26

**Authors:** Leah K. Clayton, Alexander S. Wyckoff, Sinéad M. Crotty

**Affiliations:** ^1^ Carbon Containment Lab, New Haven, CT, USA.; ^2^ Department of Earth and Planetary Sciences, Yale University, New Haven, CT, USA.; ^3^ Yale School of the Environment, Yale University, New Haven, CT, USA.

## Abstract

Climate change and fire suppression are intensifying wildfires, prompting fuel reduction treatments that will generate gigatonnes of waste biomass residues in the near term. We evaluate the potential for anoxic biomass burial to serve as a scalable approach to offtake these residues while simultaneously providing durable carbon storage. Using geospatial water balance modeling across the western United States, we find that minimum soil cover thickness varies over four orders of magnitude, reflecting strong site dependence. Life cycle emissions modeling indicates that burial is more carbon efficient than decomposition, pile burning, or biochar production. Although bioenergy with carbon capture and storage ultimately yields greater carbon benefits, infrastructure requirements and economic analysis indicate that burial is a more immediate, cost-effective option. Burial could thus provide a scalable, carbon-efficient bridge, pending further study of decay and storage controls. Combining biomass carbon removal and storage with wildfire mitigation forestry illustrates the opportunity to leverage shared underlying goals for net climate and economic benefit.

## INTRODUCTION

Climate change–induced warming and drought conditions are fueling larger and more severe wildfires globally, emitting 7 to 16 billion metric tons of carbon dioxide equivalent (MTCO_2_e) annually (*1*, *2*). This challenge is particularly pronounced in the contiguous western United States (US West), where human development in the wildland-urban interface, increased fuel aridity, and over a century of fire suppression have resulted in high-risk conditions and forests that are substantially overstocked (*3*). Ecological thinning, the selective removal of small-diameter trees and understory vegetation to decrease fuel volume and connectivity, is a first step to reducing the risk of high-severity wildfires and stewarding the safe return of historical fire regimes, cultural fire, or prescribed fire to appropriate landscapes (*3*, *4*).

The United States Forest Service (USFS) and other tribal, federal, state, and local agencies are increasing the pace and scale of ecological thinning as a part of proactive hazardous fuel treatments (*5*). Conservative estimates suggest that these activities will generate more than 1.2 billion bone-dry metric tons (BDMT) of low-value waste residues by 2032, equivalent to more than 2 billion MTCO_2_e that need to be removed from the landscape (*6*, *7*). However, few commercial markets exist for this material, and it is often in remote locations. As a result, nonmerchantable thinned material is most commonly piled and burned on site, releasing substantial quantities of greenhouse gases and criteria air pollutants while costing upward of $1800/ha (or >$50 per BDMT) (*8*–*10*). If higher-value end uses for these residues were available, then those pathways could serve to both use the inherent value of the biomass (e.g., energy content, structural characteristics, or embodied carbon) and enable more effective distribution of wildfire mitigation personnel and resources (*11*).

Biomass carbon removal and storage (BiCRS), a form of carbon dioxide removal (CDR), is a potential mechanism to valorize the removal of these nonmerchantable residues from the landscape, mitigate the emissions from decomposition or pile burning, and move carbon from the fast carbon cycle to longer-term storage reservoirs. BiCRS defines a set of pathways that use plants or algae to remove CO_2_ from the atmosphere and store it underground or in long-lived products without damaging and, ideally, while enhancing, social-environmental values (*12*). BiCRS approaches are based on the “Aines Principle,” which states that the value of using biomass for carbon removal may exceed its energy value (*12*). Therefore, while some BiCRS approaches use technologies that promote both energy and carbon storage [e.g., bioenergy with carbon capture and storage (BECCS)], other non-energy approaches focus exclusively on carbon storage as the product (e.g., biomass burial, biochar storage, and bio-oil injection) (*13*). BiCRS pathways range in their requirements and characteristics, including feedstock properties and volumes, capital and operational costs, technology and deployment readiness, infrastructure requirements, carbon efficiency, end product value, and market readiness (*6*, *13*, *14*).

This multitude of factors introduces both temporal and spatial dimensions to BiCRS pathway suitability and implementation decision-making. While prospective modeling of ideal future scenarios prioritizes the highest economic value end uses, such as biomass gasification to hydrogen with CO_2_ storage (*15*), the reality of the wildfire crisis necessitates decision-making about the best feasible use in the near term and may favor lower-technology, lower-infrastructure pathways that could be deployed sooner at lower cost. Long-term, sustainable forest management alongside the advancement of infrastructure and technological readiness may additionally favor a different set of pathways.

From a geospatial perspective, thinned residues are produced across broad areas and, often, in regions distant from infrastructure hubs or suitable geologic CO_2_ storage sites. Non-energy BiCRS pathways may be best suited for the most remote, or “stranded,” sources of waste biomass, where the biomass needs to be removed for wildfire mitigation but is too dispersed, too inconsistently produced, or too low in volume to feasibly contribute to BECCS or product markets. However, the economics for these projects may be prohibitive without favorable underlying conditions. Spatially identifying regions where the climate and natural resources are most favorable for a given BiCRS pathway proximate to where there is the greatest need for wildfire mitigation helps direct limited resources toward projects with the highest likelihood of net climate, ecosystem, and economic benefit.

In recent high-profile work, biomass burial has been proposed as a gigatonne-scale solution that can be durably deployed (1000+ years) at low cost in a globally distributed manner (*16*–*18*). These studies indicate that biomass burial could prevent decomposition and, therefore, prevent greenhouse gas emissions by relying on anoxic, dry, or cold storage conditions maintained in perpetuity (*16*–*19*). The ability to establish and maintain these conditions varies across space due to site-specific abiotic and biotic conditions and storage engineering. The inherent temporal unpredictability and scientific uncertainty of how these conditions evolve over time and the durability of storage engineering highlight the need for further investigation.

In the US West, creating and maintaining anoxic conditions is the most plausible primary mechanism to limit decay for biomass burial. Maintenance of conditions perpetually dry enough to completely prevent microbial metabolism is infeasible in soil storage environments without system engineering or additives (*14*, *16*), even in arid regions (*20*). Perpetually frozen environments suitable for burial are not present in the US West (*21*). Anoxic conditions are created and maintained by isolating stored biomass from the atmosphere and minimizing gas permeability, with either a sufficiently thick soil layer (dependent on factors such as soil type, compaction, and saturation), an engineered low-permeability membrane, or a combination of the two (*13*, *19*, *22*).

While anoxic conditions do not halt decomposition entirely, anaerobic decay pathways are slower and less energetically favorable than aerobic decay pathways (*23*). For anaerobic decomposition, once more thermodynamically favorable electron acceptors (e.g., nitrate, iron, and sulfate) are depleted, methanogenesis becomes the dominant terminal pathway (*24*). Methanogenesis produces both CO_2_ and methane (CH_4_), which has a global warming potential ∼28 times that of CO_2_ over a 100-year horizon (GWP_100_) (*25*). Limiting the rate of anaerobic decomposition is, therefore, critical, as even modest CH_4_ emissions can substantially erode or negate the potential climate benefit of biomass burial.

Under anoxic conditions, anaerobic decay pathways are limited in total extent by the chemical recalcitrance of the biomass feedstock (*26*) and in rate by abiotic storage conditions including moisture, temperature, nutrient availability, and pH (*27*). In the US West, two major factors work in favor of limiting anaerobic decomposition of buried biomass. First, softwood residues characteristic of thinning operations have low degradable organic carbon fractions (DOC_f_) vulnerable to anaerobic decay, limiting the total pool of available carbon (*28*). Second, limited water availability can strongly limit the rate of methanogenesis (*23*) as water is required for all life (*29*) and most of the US West is considered to be either arid or semiarid (*30*). Water is consumed in hydrolysis, the first and most rate-limiting step of anaerobic decomposition, where complex polymers are broken down into soluble substrates (*23*, *27*). Without sufficient water movement, intermediate organic acids accumulate and inhibit methanogenesis (*23*, *31*, *32*). Alongside limiting the total pool of carbon vulnerable to anaerobic decay, preventing water infiltration from groundwater intrusion, lateral flow, or surface percolation into an anoxic burial chamber is, therefore, a key mechanism to limit the rate of methanogenesis.

Foundational principles for biomass burial in anoxic soil environments can be found in literature for landfill design, in which waste decomposition and undesirable by-product production are controlled by engineering covers that modulate water percolation and gas permeability (*31*). In their simplest form, landfill covers can create an anoxic storage environment at depth with a sufficiently thick, compacted soil layer and rely on the water storage and evapotranspirative (ET) capacity of a vegetated soil layer to prevent water percolation into the stored waste (*31*). Further, an aerobic soil zone near the surface hosts methanotrophic bacteria that oxidize CH_4_ diffusing upward from the anaerobic storage zone, converting it to CO_2_ before it reaches the atmosphere at a fraction inversely related to the CH_4_ flux (*33*). If local conditions are suitable and storage can be maintained over time, then biomass burial with ET covers has the potential to facilitate the removal of dispersed thinned residues from high-wildfire-risk forests while providing net climate and net economic benefit.

**Fig. 1.**
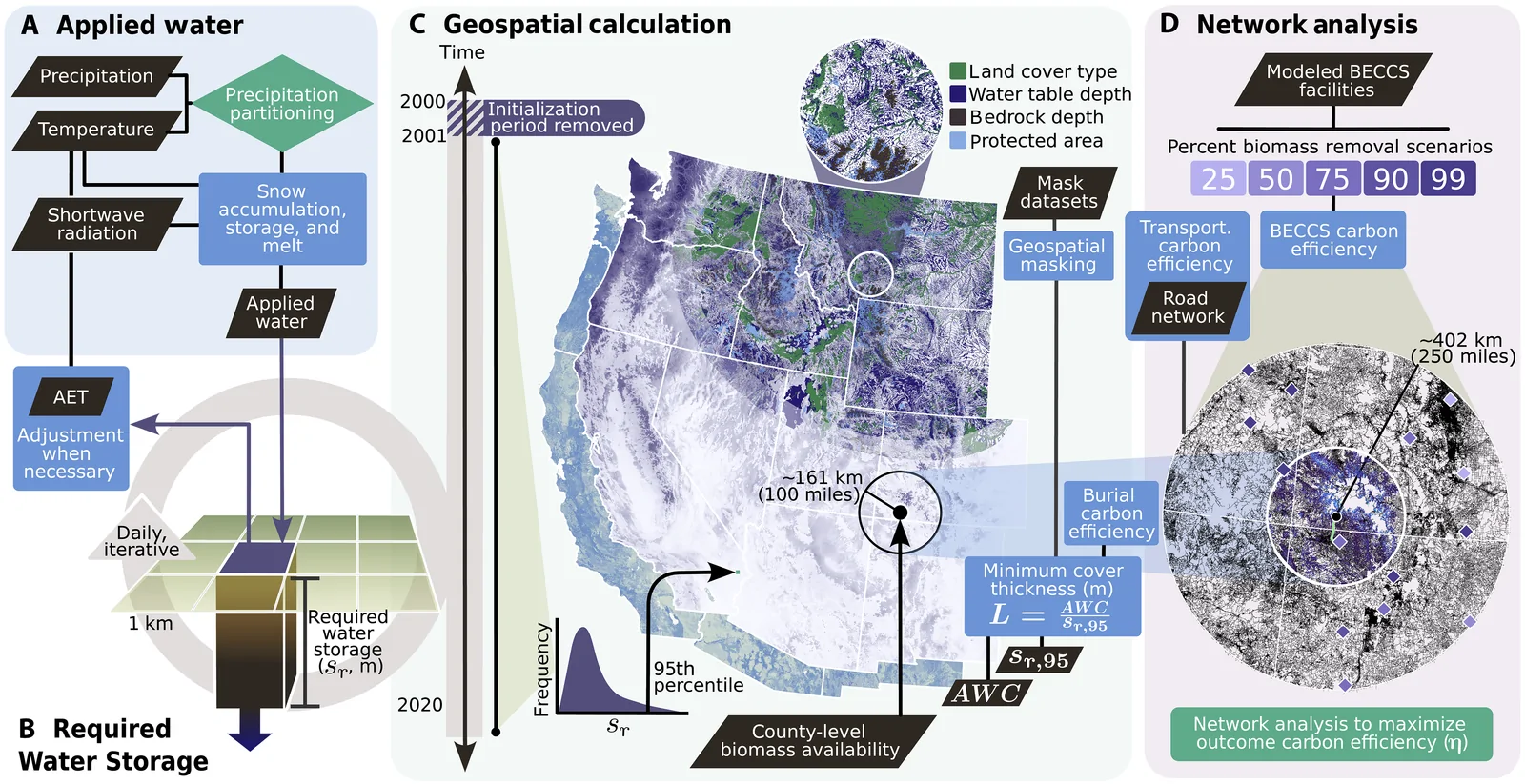
Overview of the minimum required soil cover thickness (*L*) model for biomass burial and geospatial LCA implementation The (**A**) applied water model incorporated daily, historical (2000–2020) precipitation, temperature, and shortwave radiation data (*78*) to calculate daily applied water (the sum of rainfall and snowmelt) at a 1-km resolution. Assuming an unconstrained reservoir, we calculated (**B**) required water storage (*s*_r_) iteratively on a daily time step as the water balance between applied water and AET (*79*). Using the 95th percentile highest required water storage (*s*_r,95_) for each pixel, we modeled minimum soil cover thickness (*L*) that would prevent the percolation of water to buried biomass by dividing *s*_r,95_ by soil available water content (*AWC*) (*34*) and applied geospatial masking to remove unsuitable areas (**C**). Last, we performed a geospatial, gate-to-grave life cycle assessment (LCA) for biomass burial and weighted network analysis to identify the most carbon-efficient outcomes for thinned residues. Carbon efficiency (η) of burial is then compared with counterfactual fates and alternative BiCRS end uses, including in situ biochar and (**D**) a time series of modeled BECCS scenarios (*6*).

Here, we present a mechanistically informed, geospatial assessment of biomass burial potential across the US West. We adapted an ET landfill cover planning approach (*31*) for geospatial implementation and developed a daily water balance model for the US West at a 1-km spatial resolution using meteorological data from 2000 to 2020 (Fig. 1, A and B). We calculated the minimum soil cover thickness required to prevent water percolation to stored biomass from the modeled wet-extreme required water storage and pedological data (Fig. 1C). This provides an initial geospatial quantification of site suitability from a water balance perspective, recognizing that additional requirements for anoxic storage are highly site and engineering specific.

We then analyzed the opportunity for biomass burial to serve as a carbon-efficient end use for nonmerchantable residues generated by wildfire mitigation activities in comparison with counterfactual fates (decomposition and pile burning) and alternative BiCRS fates (biochar with in situ application and BECCS). We constructed a geospatial, gate-to-grave life cycle assessment (LCA) of biomass burial for thinned residues across the US West (Fig. 1D) and used a spatial transportation network analysis to compare biomass burial with the counterfactual and alternative fates. We ultimately identify the most carbon-efficient end use for residues in high wildfire risk counties under a series of projected BECCS realizations and conduct a sensitivity analysis to highlight the key assumptions that mediate whether biomass burial is an effective CDR solution.

Given the disproportionate impact of economic considerations on project implementation and scaling, we conducted a simplified, comparative technoeconomic analysis (TEA) across potential nonmerchantable residue fates. We then leveraged all models developed herein to calculate the net climate benefit and cost associated with near-term thinning and utilization of residual biomass from high wildfire risk counties in the US West. Last, we highlight a research agenda toward context-specific, safe, and effective deployment of biomass burial, as well as the need to recognize the context dependency of all CDR pathways and mutualism between climate solutions and ecological restoration.

## RESULTS

### Modeled minimum cover thickness for biomass burial

**Fig. 2.**
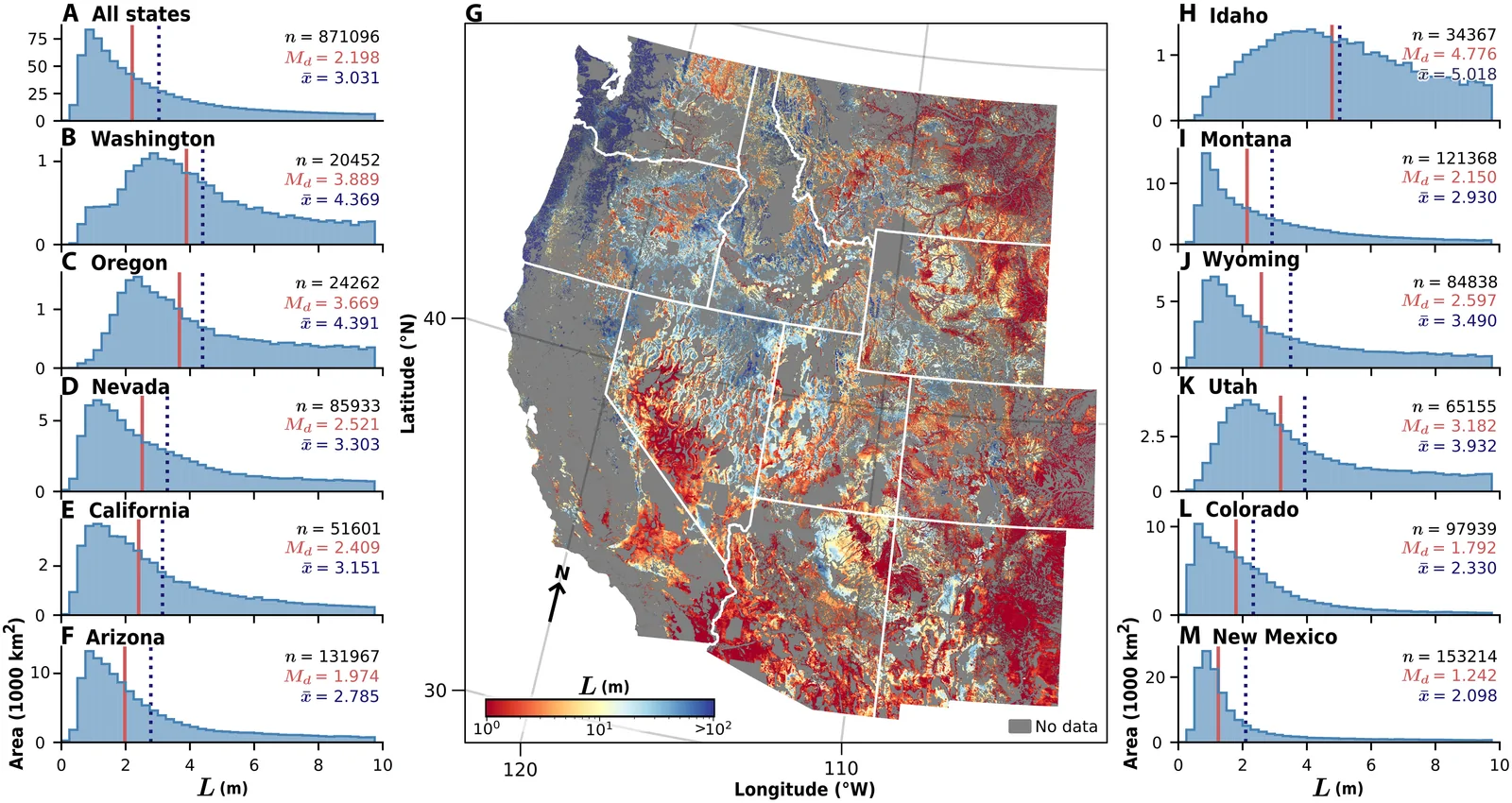
Modeled minimum soil cover thickness (*L*) for biomass burial varies over four orders of magnitude across the US West. The center map (G) displays the results of *L* (m) with a 1-km pixel resolution. Regions where pixels were removed for conditions that nullify the integrity of the modeling are displayed as “No data” (see Materials and Methods). (**A** to **F** and **H** to **M**) Histograms of *L* to a maximum of 10 m across spatial regions. The red solid line and *M*_d_ indicate the truncated distribution median, and the blue dotted line and $\bar{x}$ indicate the truncated distribution mean. (A) The aggregated distribution across all 11 states in the region of interest, and [(B) to (F) and (H) to (M)] the distributions for each of the 11 states.

Modeled minimum soil cover thickness (*L*) suitable for biomass burial with an ET cover is highly variable across the US West, ranging from 3 cm to >1 km (Fig. 2). While both extremes are unrealistic, more than 40 million ha (∼13% of land area) had *L* ≤ 2 m, suggesting substantial land area potentially suitable for burial. Regions characterized by low extremes (*L* ≤ 0.5 m, ∼0.5% land area) should be interpreted as areas most favorable for burial from a water balance perspective, although other considerations, including maintaining anoxic conditions, will almost certainly necessitate thicker soil covers. These priority regions are predominantly found in the Great Plains east of the Rocky Mountains and through the desert Southwest.

### Carbon efficiency of biomass burial

Given the geospatial *L* quantifications, availability of spatial soil carbon data (*34*, *35*), and empirically derived process emissions data (see Materials and Methods), we built a geospatially explicit, attributional, gate-to-grave LCA for anoxic burial of waste residues generated from proposed wildfire mitigation treatments across the US West. To conduct this analysis, we first aggregated modeled availability of nonmerchantable residues from hazardous fuel reduction forestry treatments at the county level (*5*, *6*), identifying 173 high-priority counties with residues across the 11 states (figs. S1 and S2). Using a network of every existing drivable road structure across the region (*36*), we optimized the spatial model to maximize carbon efficiency from each county centroid start node to burial location end nodes within a ∼161-km (100-mile) radius. Carbon efficiency (η), our output metric, is a dimensionless quantity equal to the percentage MTCO_2_e durably stored over 100 years per MTCO_2_e in the initial biomass (*37*). In other words, η reflects the percentage of the initial carbon that is durably stored, net of all process, and decay emissions (assuming GWP_100_ conversions for emissions of non-CO_2_ gases).

**Fig. 3.**
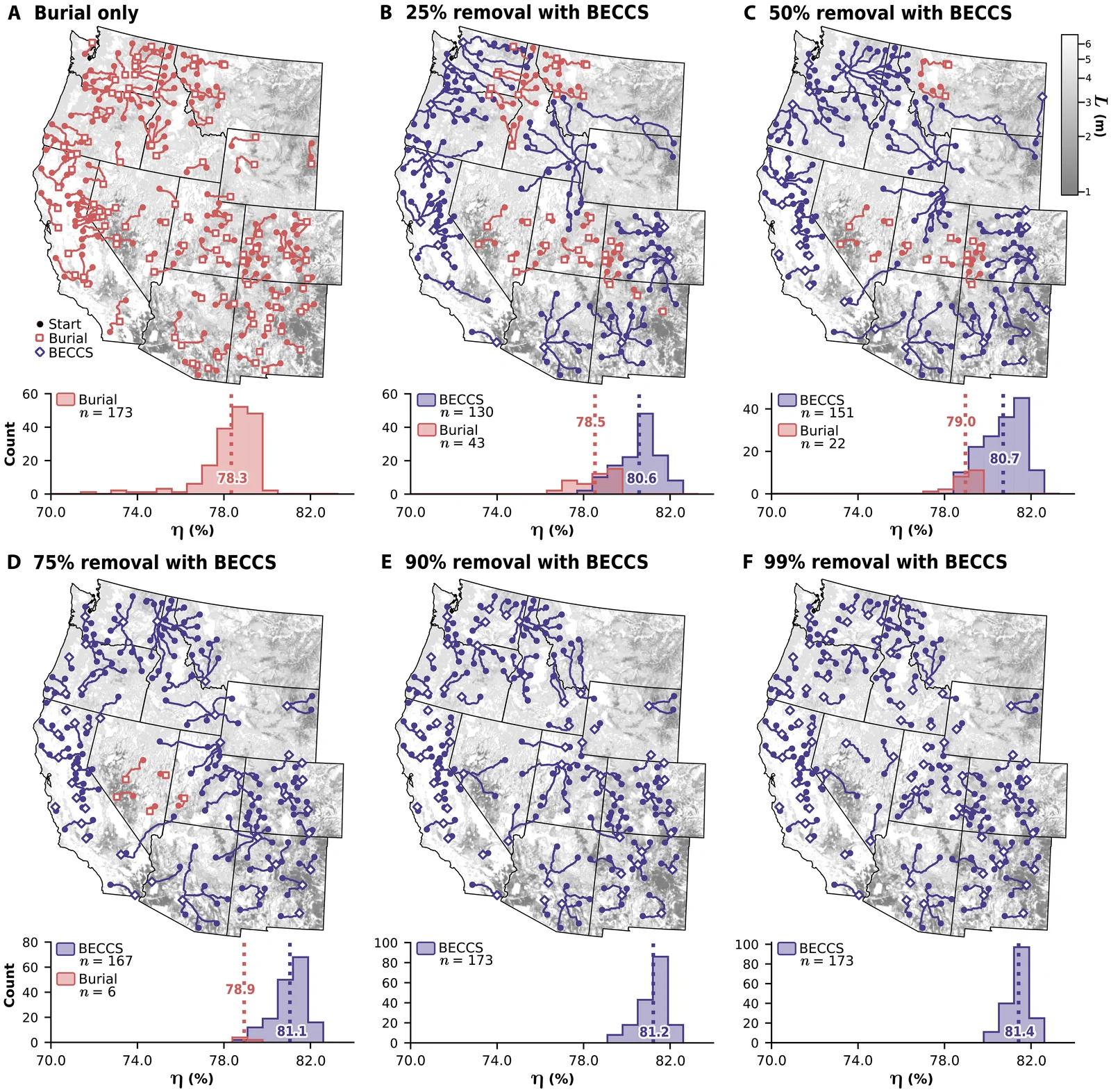
Optimized modeled carbon efficiency (η) pathways for biomass burial and BECCS over six development scenarios. Each panel displays (i) a map of modeled minimum soil cover thickness (*L*) overlain with the optimized network routes between a biomass start node (solid circle) and the most carbon-efficient end node (square for burial and diamond for BECCS) for each high-priority county modeled to have nonmerchantable residues from wildfire mitigation forestry; and (ii) a histogram of η, classified by outcome, with the labeled dotted line indicating the mean for each classification. Pathways that are optimized by burial fates are in red. Pathways optimized with BECCS fates are in blue. Each panel represents a different BECCS scenario: (**A**) a burial-only scenario with no BECCS infrastructure and (**B** to **F**) the BECCS scenarios (*6*) where increasingly more BECCS is built out based on the percentage of residual biomass removed: 25, 50, 75, 90, and 99%.

Mean η for biomass burial across all counties is 78.3 ± 1.2% (means ± 1 SD, here and following) (Fig. 3A), with one-way transportation distance of 140 ± 69 km. Most of emissions associated with biomass burial result from anaerobic decay of buried biomass over 100 years (decreases η by 18.9%, here and following), while forestry processes (0.7%), storage chamber engineering (0.8 ± 0.7%), soil disturbance (0.9 ± 0.4%), and transportation (1.0 ± 0.5%) are all minor contributors to the reductions in η for this pathway. Even in circumstances of transportation exceeding 500 km, burial η in all counties was far higher than natural decomposition (η = 6.1%), pile burning (η = 4.5%), or biochar via slow pyrolysis with on-site application (η = 43.5%). We highlight that economic thresholds, and not emissions, are likely to be the limiting factor controlling feasible transportation distances, and we revisit economic considerations in the TEA.

Biomass burial *L* affects the storage chamber engineering and soil disturbance emissions in the LCA. For the optimized burial end nodes selected in the network analysis, these contributions result in minor combined deductions to η (1.7 ± 1.1%) due to low required cover thicknesses (0.55 ± 0.47 m). However, out of all burial end nodes available to the network analysis [*L* ≤ 6.67 m and distance to existing roads of <804.7 m (0.5 miles)], storage chamber engineering and soil disturbance emissions reduce η by a combined 6.9 ± 6.5%. Combined deductions ranging up to 280% highlight that thick required covers in soils with high carbon content can result in net climate detriment, further illustrating the importance of site selection in practice.

Mean *L* of end nodes selected by the network analysis was less than the >1- to 2-m depth often required to reach the depth of oxygen extinction (*38*) without modifying the in situ soil environment or in the absence of other engineering. Therefore, we ran an additional network analysis with a minimum *L* of 1 m to understand how a theoretical threshold for thicker required soil covers would affect LCA outcomes. For this scenario, if any pixel had an *L* < 1 m, then it was set to 1 m for LCA calculations. This lowered η of selected burial end nodes by 0.5 ± 0.5% while increasing *L* by 0.52 ± 0.26 m. This decrease in average η is less than the ∼2.5% decrease expected from the per-meter deductions calculated above for the original burial end nodes. The higher emissions associated with thicker covers under the minimum *L* constraint are somewhat compensated by selecting end nodes with shorter transportation distances (one-way, −28 ± 0.002 km) and lower soil carbon content. As the depth of oxygen extinction is highly site and engineering specific and soil cover thickness is the primary mechanism used to establish requisite anoxic storage conditions in this LCA, we revisit these considerations and the need for higher resolution modeling in Discussion.

### Optimizing carbon efficiency over time

As BiCRS approaches evolve, higher value uses of biomass will be prioritized, such as BECCS (*15*). However, very few BECCS facilities have been implemented (*39*, *40*). Therefore, instead of focusing solely on preexisting facilities, we analyzed prospective BECCS facilities derived from the biocarbon infrastructure, logistics, and transportation (BILT) model (*6*) across five scenarios. These scenarios represent the percentage of biomass removed relative to the total biomass available to be removed: 25, 50, 75, 90, and 99%. We treat these scenarios as a proxy for time that identifies where higher value end uses are likely to come online in the near term and over a time frame of unspecified length, dependent on the construction of supply chains, infrastructure, and markets. To enable a geospatially informed selection of the most carbon-efficient end use of biomass under each scenario, we calculated η for BECCS facility end nodes and conducted a network analysis for transportation emissions to optimize the path from each county start node to BECCS facilities available within a ∼402-km (250-mile) radius. For each county and scenario, we queried whether BECCS, if present, or biomass burial had the higher η, indicating the most carbon-efficient outcome (Fig. 3).

Mean η for BECCS across all scenarios is 80.9 ± 0.9%, excluding the effects of any displacement of fossil product emissions resulting from BECCS. Emissions are contributed by forestry processes (decrease η by 0.7%, here and following), biomass transportation (1.3 to 5.7%), biomass preprocessing (shredding, 1.7%; and drying, 5.9%), carbon capture and storage (5.0%), and CO_2_ transport and storage (4.4%) (*37*, *41*). As the percent removal scenario increases, mean η of outcomes marginally increases as a result of shorter transportation distances. Counties without BECCS facilities in the prescribed radius in the 25 and 50% scenarios are predominantly in Idaho, Montana, and Utah, but all counties can reach BECCS facilities by the 75% scenario.

In scenarios where both BECCS and burial exist within the set radius, BECCS is found to be optimal 93% of the time. In the few cases where burial is found more carbon efficient than BECCS, the difference in η is negligible (0.4 ± 0.4%). However, the transportation distance to BECCS is 9.7 ± 8.9 times higher than to burial, indicating the importance of additional logistical and economic analyses for decision-making. Counties where burial η exceeds that of BECCS are predominantly in Colorado, Utah, and Nevada.

### Sensitivity of burial carbon efficiency to feedstock composition and storage conditions

**Fig. 4.**
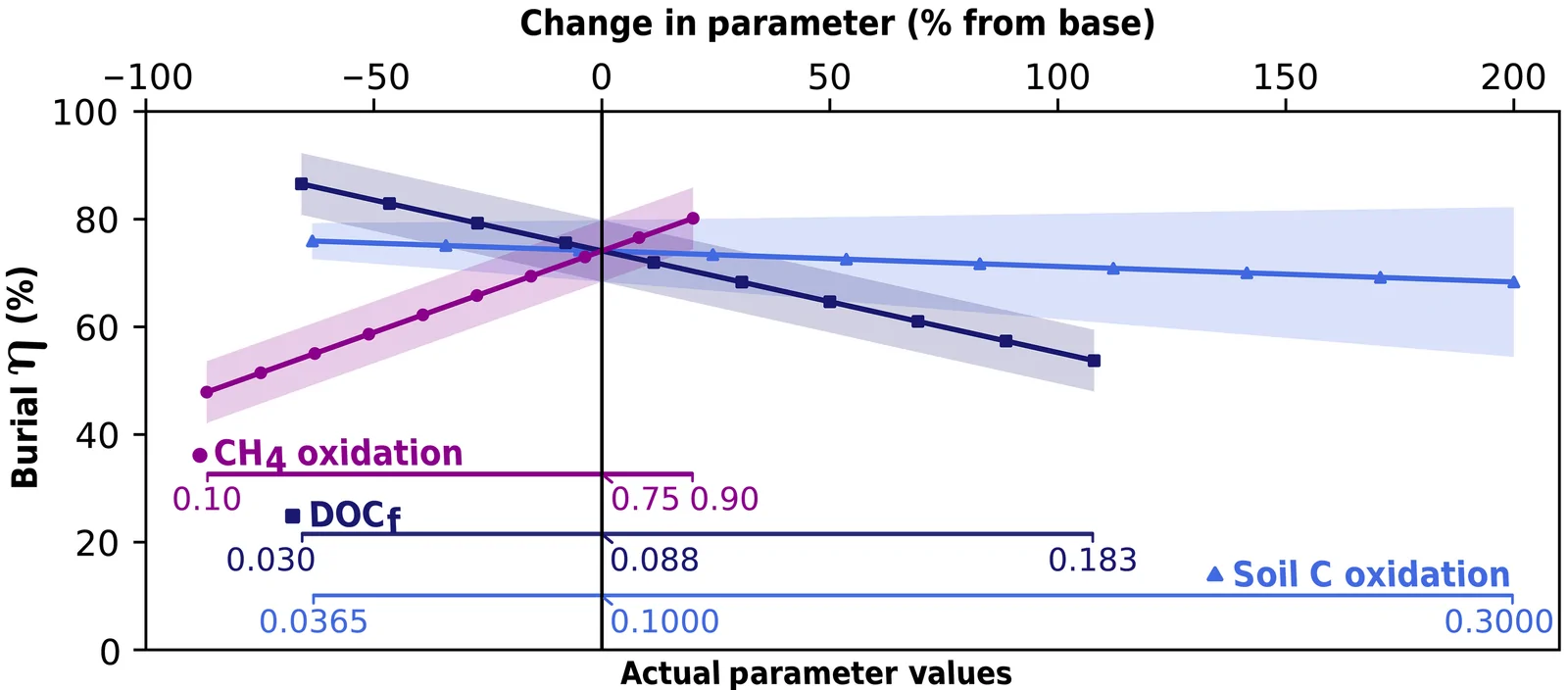
Burial carbon efficiency (η) is sensitive to feedstock degradable fraction of organic carbon (DOC_f_), cover methane (CH_4_) oxidation rate, and soil carbon oxidation rate upon excavation and construction (soil C oxidation). For each of the parameters of interest, the LCA was conducted with 10 values across the feasible range of the parameter value (colored horizontal line) while the other parameters were held constant at the base case (see table S1 for citations justifying ranges). The base case assumes the CH_4_ oxidation rate in the cover is 0.75 (*33*, *116*), feedstock DOC_f_ is 0.088 (*26*), and the soil C oxidation rate upon excavation and construction is 0.10 (*115*). The percent change in parameter from base case and resulting spatially averaged burial η (excluding transportation) pairs are plotted with a solid line indicating the linear relationship and shading indicating ±1 SD from the spatial mean.

Until BECCS facilities and supply chains are constructed, biomass burial emerges as the most carbon-efficient end use assessed herein for waste residues. However, η of anoxic biomass burial is highly sensitive to the LCA parameterization. We performed a sensitivity analysis on the three most influential parameters: (i) feedstock DOC_f_, which controls the fraction of the feedstock available for anaerobic decomposition; (ii) CH_4_ oxidation rate in the soil cover, which controls whether CH_4_ generated during methanogenesis enters the atmosphere as CH_4_ or CO_2_; and (iii) the oxidation rate of soil carbon during excavation and construction, which controls up-front losses of soil organic carbon as CO_2_ (Fig. 4).

The specific parameterization of these values, primarily DOC_f_ and cover CH_4_ oxidation rate, meaningfully changes the network outcomes. For example, the most carbon-efficient scenario (Fig. 4), a low DOC_f_ of 0.03 [softwood; see (*42*) and citations within], has a mean η of 90.7 ± 1.2% across the optimal end nodes selected in the network analysis, resulting in all counties routing to burial end nodes under all BECCS scenarios (fig. S3). Conversely, the least carbon-efficient scenario (Fig. 4), an inefficient cover CH_4_ oxidation fraction of 0.10, results in a mean η of 52.1 ± 1.2% across the optimal burial end nodes selected in the network analysis (fig. S4). In this scenario, BECCS is always selected over burial when BECCS facilities are available. While burial remains significantly more carbon efficient than decomposition or pile burning, the carbon efficiencies under the low CH_4_ oxidation parameterization fall near those modeled for biochar (η = 43.5%). The LCA is less sensitive to the parameterization of the oxidation rate of soil carbon than to the magnitude of soil carbon available, which is geospatially determined.

### Technoeconomic analysis of BiCRS pathways

BiCRS pathway η is critical for understanding potential net climate benefit, but project implementation and scale-up is contingent on economics. Depending on the relevant factors for each counterfactual and BiCRS pathway, project net income is a function of the cost of forest processes, biomass transportation costs, capital expenditure (CapEx), operational expenditure (OpEx), carbon credit yield and price, and salable product yield and price. We performed a simplified TEA to calculate net income (United States dollar, $, 2026) per MTCO_2_e in the initial biomass (MTCO_2_e_i_) for pile burning, burial, BECCS, and biochar as a function of these component costs. We established a base-case scenario from current market prices and costs in published literature and used the mean transportation distances and carbon efficiencies from the LCA in this study for burial and BECCS (table S2). We identified a range of lower and upper bounds from published literature and this study to create a parameter space to model uncertainty in parameter values over time and under different conditions in a sensitivity analysis (fig. S5).

Pile burning returns a net loss of −$49 MTCO_2_e_i_^−1^ under the base case and operates at a net loss across the entire parameter space, ranging from −$28 MTCO_2_e_i_^−1^ to −$80 MTCO_2_e_i_^−1^. The two component costs—forest processes (felling, skidding, and piling; included in every pathway) and burning—each contribute approximately half of the cost of the pathway.

In contrast, all BiCRS utilization pathways return a net profit under the base-case scenario, assuming a carbon credit price of $100 MTCO_2_e^−1^ (*6*). Burial returns the highest net profit of $21 MTCO_2_e_i_^−1^, followed by BECCS at $12 MTCO_2_e_i_^−1^ and biochar at $1 MTCO_2_e_i_^−1^. Introducing a green hydrogen tax incentive (*43*) for BECCS, otherwise under the base-case scenario, raises net profit to $197 MTCO_2_e_i_^−1^. Biochar production with on-site application (hereafter, in situ biochar), as modeled in the LCA component of this paper, assumes no salable product revenue and returns a net loss of −$22 MTCO_2_e_i_^−1^.

Profitability across all BiCRS pathways is most sensitive to carbon credit price, followed by salable product price for BECCS and biochar. Under the most favorable combination of all parameters, including a $300 MTCO_2_e^−1^ carbon credit price, BECCS and biochar achieve net incomes 111 and 39% higher than burial, respectively (BECCS, $508 MTCO_2_e_i_^−1^; biochar, $336 MTCO_2_e_i_^−1^; and burial, $241 MTCO_2_e_i_^−1^). In situ biochar would only reach a maximum net income of $79 MTCO_2_e_i_^−1^, less than a third that of burial. Given the close relationship between η and carbon credit generation, economic feasibility is also highly sensitive to η. This is particularly true for burial, where carbon credits are the sole income source.

Following market factors, BECCS and biochar are most sensitive to OpEx, and both the OpEx and CapEx of these pathways are several times higher than that of burial. BECCS and biochar OpEx per MTCO_2_e_i_ is 4.7 and 6.9 times that of burial, respectively. Similarly, BECCS and biochar CapEx per MTCO_2_e_i_ are 7.8 and 3.2 times that of burial, respectively. However, this analysis excludes the monitoring, reporting, and verification (MRV) costs associated with receiving carbon credits that may be more notable for burial than the other pathways.

Biomass transportation cost and distance exert a comparatively modest influence on overall pathway economics. Under the base-case assumptions for trucking in the US West, biomass transportation costs ∼$0.12 per metric ton of green biomass per km (*44*), equivalent to $10 MTCO_2_e_i_^−1^ 100 km^−1^. However, while the TEA is not highly sensitive to transportation cost or distance, biomass transportation costs are prohibitive if there is no profit associated with the end use of the material (*45*).

### BiCRS opportunities with wildfire mitigation in the US West

Residual biomass from near-term fuel reduction treatments in the US West could offer an immediate opportunity to direct biomass to more carbon-efficient and cost-effective BiCRS pathways in contrast to the emission-intensive, costly practices of pile burning. In the 2026 fiscal year budget, the newly created US Wildland Fire Service proposed treating ∼1.42 million ha [3.5 million acres (ac)] for hazardous fuel reduction across the United States (*46*). This represents 1/20th of the ∼28.3 million ha (70 million ac) proposed to be treated over 10 years in the US West alone (*5*, *6*).

We calculated the potential net climate and economic outcomes associated with allocating nonmerchantable residues from the treatment of 3.5 million ac to pile burning (counterfactual) versus BiCRS pathways (burial, BECCS, and biochar) as modeled in the base-case LCA and TEA. We selected nonmerchantable residues from the highest wildfire hazard counties in the US West (*6*, *47*), restricted to accessible terrain [within 152 m (500 feet) of roads, slopes of <20%] and adding counties in order of decreasing wildfire hazard potential until the 3.5 million ac target was reached. In total, this represents 43 million BDMT of nonmerchantable residues from 57 counties (fig. S6).

For the modeled acreage, required forestry processes (felling, skidding, and piling biomass) are estimated to cost $1.3 billion. As we assume that all pathways require thinning and aggregation, this cost is incurred for all pathways. In the counterfactual scenario, burning the assembled piles would cost an additional $1.8 billion, resulting in a net cost of −$3.1 billion. Burning of these residues would additionally emit ∼75 million MTCO_2_e and >260,000 metric tons of PM_2.5_ to the atmosphere (*48*).

Using the modeled LCA outcomes and TEA base-case equations developed herein (including a carbon credit price of $100 MTCO_2_e^−1^), both burial and BECCS result in net profit of similar magnitudes. Allocating all material to burial resulted in the storage of ∼61 million MTCO_2_e (mean η = 77%) and a net profit of $2.0 billion. Allocating all material to BECCS with base-case salable product revenue ($102 MTCO_2_e^−1^) (*6*) as well as carbon credit revenue resulted in a similar, but slightly higher, modeled storage of ∼64 million MTCO_2_e (mean η = 80%) and a net profit of $2.1 billion. With tax incentives [e.g., for green hydrogen; (*43*)] or lucrative product markets for BECCS, this profit could increase multifold, >40× according to this analysis. However, this is under the 90% removal scenario where BECCS outcomes are available and most carbon-efficient for all counties (Fig. 3E). Five of the 57 highest-priority counties selected do not have modeled BECCS facilities in the prescribed radius under the 25% removal scenario. In situ biochar was modeled to durably store 34 million MTCO_2_e (mean η = 44%) and emit >23,000 metric tons of PM_2.5_ at a net cost of −$2.6 billion. The sale of the biochar at current market rate would result in a modeled net profit of $780 million but require product transportation which is not accounted for. While both burial and BECCS perform better than biochar and result in similar modeled net profit and carbon storage, these BECCS facilities are not now available.

## DISCUSSION

As wildfires grow larger and more destructive under a warming climate, we investigated whether BiCRS could support mutually beneficial solutions to mitigate severe wildfire risk and provide CDR. Of the available BiCRS pathways, biomass burial offers the most immediate, low-infrastructure route to durably store carbon from nonmerchantable residues in the US West at a high carbon efficiency. However, this is entirely dependent on anoxic storage chambers being prudently sited, designed, implemented, and maintained, and site suitability varies widely across the region. Despite this, all high-priority counties for wildfire mitigation forestry can reach locations suitable for burial with a high average η of 78.3%, similar to that of prospective modeled BECCS facilities (η = 80.9%). These BiCRS outcomes are significantly more carbon efficient than biochar (η = 43.5%) or the business-as-usual scenarios for nonmerchantable residues: Pile burning (η = 4.5%) or decomposition on site (η = 6.1%). BiCRS pathways offer the potential to durably store carbon from these residues at net profit, whereas ecological thinning has historically been hindered by the substantial cost of forestry and pile burning, as well as logistical challenges (*10*, *49*, *50*). Project economics are critical for the feasibility and scaling of any approach, and we find a strong dependency of profitability on carbon credit pricing and salable product markets. Our preliminary comparative analysis in the US West found that allocating nonmerchantable residues to burial or BECCS from acreage that could be treated in a single year could result in a net profit of the same magnitude of the entire US Wildland Fire Service annual budget (*46*) while storing >60 million MTCO_2_e and preventing the release of >260,000 metric tons of PM_2.5_. None of our analyses herein quantify the avoided climate, socioeconomic, or environmental damages associated with reducing severe wildfire risk, and including these factors would likely only increase the favorability of optimal BiCRS pathways (*45*).

However, moving from theoretical modeling to on-the-ground reality highlights the need for substantial additional work. Scaling BiCRS from analysis to practice will require expanding labor markets and forestry capacity in regions that previously have not supported major forest industry, developing transportation networks and logistics for dispersed biomass feedstocks, building processing and storage infrastructure, and cultivating the public trust and political will necessary for long-term land use commitments (*51*), all while upholding sustainable feedstock sourcing principles and “do no harm” ethics inherent in BiCRS (*12*). BECCS facilities and corresponding infrastructure have not yet been built at scale, and the feasibility of large-scale deployments remains contested, with concerns spanning unproven technology integration, high capital costs, inadequate carbon pricing or policy frameworks, and limited social acceptance (*39*, *40*, *52*). Judicious biomass burial could provide a potential bridge strategy between near-term emergency response to facilitate urgent wildfire risk mitigation and long-term, higher-value end uses, such as BECCS. Yet, we found that the potential for biomass burial is highly site dependent and relies on careful storage chamber construction and maintenance in perpetuity to be durable carbon storage. Our findings point to three key considerations for advancing responsible solutions: (i) addressing near-term research priorities to promote the durability and safety of biomass burial; (ii) understanding the fundamental context dependency of CDR performance that necessitates science-driven, place-based deployment strategies; and (iii) leveraging inherent mutualisms between climate solutions and ecological restoration to address the dual anthropogenic crises of wildfire and climate change.

### Research frontiers and considerations for biomass burial

Preexisting abiotic and biotic, including anthropogenic, conditions act as first controls on site suitability for biomass burial, determining both baseline feasibility and implementation requirements. We find that *L* varies across four orders of magnitude across the US West. The model for *L* is driven by water balance conditions and soil water storage capacity, with geospatial masking of conditions that would preclude successful anoxic burial. Some of these factors are physical constraints—such as shallow bedrock depth, shallow water table depth, or surface water—while some incorporate human decision-making and value systems—including protected land status and the exclusion of highly developed or irrigated lands (*31*). While *L* is not directly a function of soil organic carbon content, the LCA illustrated that it should be considered for site selection: Emissions from the oxidation of soil carbon during excavation to depths of <10 m can be substantial enough to cause net climate detriment. Prudent site selection will need to account for both abiotic forcing and biotic considerations, as well as the multistakeholder, multifold decisions inherent in ecosystem disturbance, project implementation, and long-term stewardship (*53*). Aside from site selection, research frontiers around storage chamber design, long-term maintenance, and feedstock characterization highlight further need for interdisciplinary research to facilitate responsible biomass burial where appropriate.

Durable biomass burial is fundamentally dependent on establishing and maintaining conditions inhospitable to decomposition in perpetuity (*13*, *16*–*18*). While limiting water infiltration limits the rate of anaerobic decomposition, for anoxic burial with a soil ET cover, storage chamber design and maintenance must modulate gas permeability and risk of disturbance to maintain oxygen-free conditions, in addition to water balance considerations (*31*). Gas permeability of the soil cover controls oxygen intrusion and decay product emission, and it is a function of site-specific diffusion gradients, biota, soil type, soil saturation, and soil compaction, all of which can evolve over time. From an implementation perspective, thinner covers are desirable to reduce soil disturbance, ecosystem disruption, excavation costs and emissions, and other logistical constraints (*42*). However, the cover must be thick enough and designed in a manner to reach oxygen extinction at depth for anoxic storage conditions (>1 to 2 m) (*38*), shed or store water to prevent percolation (*23*, *54*), support a sufficient aerobic zone for vegetation and methanotrophic activity (*17*, *18*, *42*, *55*), and resist erosion or other disturbances (*31*).

In water-limited regions, the minimum cover thickness indicated by *L* is likely to be too thin to meet these constraints without modifying the soil environment (i.e., compaction) or introducing engineered barriers. Whether engineered barriers common in conventional landfill design, such as low permeability clay or synthetic layers, are warranted merits further investigation into an important design trade-off between potential improvements in long-term performance and added cost, construction complexity, and embodied emissions (*31*). Detailed modeling [e.g., (*56*, *57*)] with site-specific data should be performed for any proposed burial to understand gas diffusion, microclimate, high-resolution edaphic conditions, and proposed storage chamber engineering. Designing covers that can balance all criteria, as well as meet temporal challenges such as managing water balance before a mature vegetative cover is established or maintaining cover integrity in perpetuity, represents a key frontier for burial.

From an MRV perspective, predisturbance and continuous in situ monitoring of gas fluxes will be essential to understand gas permeability and demonstrate carbon storage. This is critical as soils have existing background gas fluxes that need to be accounted for [e.g., predisturbance CH_4_ uptake in arid soils; (*58*)], and cover degradation over time has been noted in the landfill literature (*32*). While CH_4_ production in the storage chamber should ideally be limited to the greatest extent possible, results of the burial LCA sensitivity analysis underscore the urgency of reducing uncertainty around the CH_4_ oxidation rate in a soil cover. Previous work indicates high CH_4_ oxidation rates are correlated with low CH_4_ fluxes (*33*), and isotopic analysis of CH_4_ oxidation rates (*59*) and methanotrophic biocovers (*60*) represent promising research opportunities. Last, we highlight the need for improved standardization and context-specific validation of feedstock DOC_f_ estimates to quantify the carbon pool available for anaerobic decay (*13*, *28*, *61*).

In the long-term, future changes in climate will strongly influence both the feasibility and durability of biomass burial. The analysis presented here uses historical meteorological data from 2001 to 2020. In the US West, these years have been identified as the inception of megadrought (*62*). While future climate projections from Earth systems models could have utility for understanding the spatial and temporal evolution of possible conditions under different emission scenarios, current challenges indicate that these products are not suitable for prescriptive use. These include the overestimation of atmospheric water availability in arid regions (*63*) and persistent biases for phenomena such as the North American Monsoon (*64*), in addition to the uncertainty of anthropogenic action to either address or exacerbate climate change. Yet, thermodynamics and recent observations can inform initial predictions for future conditions. More energy in the atmosphere resulting in predominantly warmer temperatures will increase atmospheric water vapor holding capacity and thus precipitation and evapotranspiration extremes, with both wet and dry hydrological extremes manifesting across event magnitude, spatial coverage, and duration (*65*). Simultaneously, climate change will continue to alter fire risk (*1*), management options (*66*), and postfire trajectories (*67*). Design, modeling, and site selection for durable burial must be informed by these extreme scenarios and their potential evolution over time.

### Context dependency of CDR and project siting

The success of all CDR approaches is inherently context dependent. Performance hinges on how local abiotic, biotic, and anthropogenic conditions interact with the underlying CDR mechanism or technology, and these should be assessed at the site-level [e.g., (*68*–*70*)]. Each BiCRS pathway—including energy BiCRS (BECCS) and non-energy BiCRS (such as biomass burial, biochar, bio-oil injection, and mass timber)—has a unique suite of characteristics (*13*). These characteristics include carbon efficiency (and the factors that affect it), CapEx and OpEx, transportation requirements, suitable biomass feedstock properties or volumes, technology or commercialization readiness, infrastructure requirements, salable product value, market readiness, and sociopolitical acceptance. As these BiCRS approaches are considered for deployment, the match between characteristics and contexts needs to be evaluated as the abiotic, biotic, and anthropogenic landscape varies over both space and time.

This principle of context dependency extends well beyond BiCRS. Across CDR pathways, the performance of individual approaches is governed by local conditions in ways that can produce variance spanning orders of magnitude, including for enhanced rock weathering (*70*), soil carbon sequestration (*69*), and ocean alkalinity enhancement (*68*). Deployment strategies should be guided by rigorous, site-specific assessment of the conditions that control performance. The temptation to deploy approaches broadly based on favorable average performance estimates or laboratory results risks both wasted resources and unintended consequences. Spatially explicit LCA and TEA, integrated within network-based modeling frameworks, provide foundational tools for matching CDR approaches to landscapes where evidence indicates that they are most likely to be effective, durable, and safe.

Beyond existing conditions, the biophysical conditions that control CDR performance are themselves nonstationary. Forest processes—including growth rates, mortality, and species composition—will shift with changing climate, disturbance regimes, and management priorities [e.g., (*67*, *71*)]. Fire risk will evolve in response to fuel accumulation and moisture, fuel treatment and fire history, weather and climate, and human decision-making (*1*, *3*, *72*). Simultaneously, the business-as-usual scenarios used to assess the counterfactuals for CDR will evolve (*3*, *49*). All these factors highlight an imperative for a time dimension in modeling and continuous reassessment of approaches and crediting over time (*73*). Rigorous, ongoing MRV is paramount for detecting performance changes, validating durability claims, and triggering adaptive management responses.

### Mutual climate solutions and ecological restoration

The convergence of wildfire risk reduction and BiCRS represents a compelling instance of mutual solutions, in which the combination of the two projects provides greater scale or climate benefit than either activity could achieve alone. In the US West, by using residues from hazardous fuel treatments for BiCRS pathways, the economic equation could be fundamentally altered, transforming a costly waste product into a feedstock for durable carbon storage and, potentially, into a revenue-generating asset through carbon or product markets. Critically, if these analyses were to include the avoided damages from wildfire risk reduction (e.g., preservation of ecosystem services and avoided suppression costs, carbon and PM_2.5_ emissions, health costs, property damage, and infrastructure losses) or the displacement of fossil carbon emissions from BECCS, then the net climate and net economic benefits would be substantially greater than those reported here [e.g., (*14*, *45*, *74*)]. Accounting for these cobenefits would further support the favorability of BiCRS outcomes compared with current counterfactuals.

This wildfire-CDR mutualism in the US West can be understood as an example of a broader principle. Many existing ecological restoration and resource management needs are underfunded. Simultaneously, at current carbon credit prices, the economics of CDR alone are unlikely to sustain gigatonne-scale deployment for most approaches (*52*). Neither challenge can support scaled deployment on its own, but when paired, the economics could shift. Carbon revenues reduce the net cost of restoration work that would, otherwise, depend on public appropriations, while the management activity generates feedstock, site access, and operational infrastructure that CDR would, otherwise, have to build and finance from scratch. In the US West, instead of fragile systems dependent on a single revenue stream, we propose that these mutualisms enable one in which wood product sales, carbon credit revenues, public restoration funding, and the avoided costs share the burden that neither ecological restoration nor CDR could bear alone. Ultimately, addressing environmental degradation and advancing carbon removal are not separate challenges but intertwined components of the same system. Leveraging underlying shared goals and grounding implementation in site-specific science coupled with rigorous verification can help move both ecological restoration and CDR toward more adaptive, evidence-based, and climate-resilient practices.

## MATERIALS AND METHODS

### Minimum soil cover thickness (*L*) modeling

#### ET cover theory

Modern landfill covers are designed to limit the movement of gases and liquids into and out of stored waste to minimize decomposition and environmental contamination, as outlined in *Water Balance Covers for Waste Containment* by Albright *et al.* (*31*). This includes minimizing leachate or gas emissions that may contain carbon decay products such as carbon dioxide (CO_2_) and methane (CH_4_). Conventional landfill covers primarily use synthetic hydraulic barriers or compacted clay layers to limit the saturated hydraulic conductivity of the cover, the key regulatory metric in the United States. Conventional landfills consider the local soil water balance with revegetation after landfill closure, but the primary mechanism for minimizing percolation is the low-conductivity layer. ET covers are alternative cover designs that do not use synthetic barrier layers or compacted clay layers. Instead, ET covers prevent the percolation of water to stored waste by (i) relying on the temporary water storage capacity of near-surface soils to hold water when precipitation exceeds evapotranspiration and (ii) the subsequent removal of water via evaporation and transpiration, controlled by atmospheric demand and vegetation. ET covers are generally cheaper to implement and have lower embodied carbon than conventional covers because they avoid the production, transportation, and installation of the hydraulic barrier layer. Landfill ET covers have been implemented successfully, especially in semiarid and arid regions, and they are plausible mechanisms to maintain conditions inhospitable to decay in regions without notable clay deposits, like much of the US West (*75*), or when synthetic hydraulic barriers are otherwise undesirable.

As discussed in Introduction, we focus on water balance for geospatial modeling because it is critical to limit the rate of anaerobic decomposition. In the context of burial as a BiCRS approach, the goal is to maximize carbon storage over time by minimizing the extent and rate of decomposition, which are a function of both biomass composition and storage conditions. Limiting water intrusion into waste stored under anoxic conditions limits decomposition by several mechanisms. Anaerobic decomposition in landfills is microbially mediated, and water availability is required for all life (*29*). Hydrolysis is critical for both aerobic and anaerobic decomposition to transform complex polymers to soluble sugars, amino acids, and other products (*23*, *27*). It is also the first and slowest step of anaerobic decomposition, and limited water availability can significantly reduce the rate of anaerobic decomposition (*23*). Under unsaturated conditions, biotic processes take place in thin films of water surrounding particles, and, without the movement of water to transport and dilute the products, organic acids can accumulate and prevent methanogenesis (*23*).

Water balance conditions are the primary consideration when designing ET covers, and we calculated preliminary soil cover thickness following the guidance of Albright *et al.* (*31*). This approach excludes several abiotic factors such as temperature, redox conditions, and gas diffusion that are important controls for decomposition in biomass burial but cannot be assessed using the geospatial framework developed here as they are highly site or engineering specific. As temperature influences all reaction kinetics, increases in ambient soil temperature may increase the rate of both methanogenesis and methanotrophy, although the sensitivity of microbial communities to changes in environmental conditions needs further study (*76*). Oxygen extinction depth and CH_4_ oxidation in the soil cover are a function of site-specific soils and biota and cover compaction and engineering (*77*). These gas diffusion factors are critical for maintaining anoxic conditions and promoting net climate benefit for biomass burial, and, as such, they should be carefully analyzed for any proposed project. However, they will require site-specific characterization and are, therefore, out of scope of this study (with the exception of a sensitivity analysis performed on the LCA, see the “Biomass burial LCA sensitivity analysis” section). We highlight that if a sufficiently thick soil cover is the primary mechanism to maintain anoxic conditions, then this required cover thickness determined by gas permeability will likely exceed that modeled from water balance in water-limited regions. Detailed site-specific, depth-parameterized modeling (e.g., HYDRUS and UNSAT-H) (*56*, *57*) should be performed with in situ, high-resolution data for expected and extreme scenarios to further understand the implications of local weather and climate, edaphic conditions, storage chamber engineering, biotic intrusion, and proposed revegetation. Herein, we use in situ soil properties (*34*), precipitation (*78*), and actual evapotranspiration (AET) (*79*) quantified geospatially across the US West to calculate minimum required soil cover thickness of a monolithic ET cover given the existing, unmodified soils and near-historical climate conditions.

The total water storage capacity (*S*_c_) of a given soil is equal to the depth-integrated volumetric soil water content at field capacity (θ_c_), the amount of water a layer of soil can hold up to the point of percolation to a greater depth. By convention, field capacity water content is measured at 33-kPa suction. Available water storage capacity (*S*_a_) is defined as the depth-integrated total storage capacity minus the water content that cannot be accessed by plants, defined by the volumetric water content at minimum storage, θ_m_, also known as the wilting point. *S*_a_ over depth *L* is reasonably approximated by

$$S_{a}=\int \left(\theta_{c}-\theta_{m}\right)\mathrm{dz}≅\left(\theta_{c}-\theta_{m}\right)L$$ (1)

Wilting point is conventionally measured at 1500-kPa suction although there is evidence suggesting that the true wilting point for plants in arid and semiarid climates can be higher due to plant adaptation to dry conditions (*80*, *81*). At suctions greater than the wilting point, the water content of the soil is low enough that capillary action and, thus, root water uptake become impossible.

Given that the goal of an ET cover is to prevent the percolation of water to stored biomass, the available storage capacity needs to be greater than or equal to the required water storage (*s*_r_), often termed root-zone water storage capacity, as determined by the local climate. Equation 1 can be rearranged and combined with this constraint to calculate the minimum required soil depth (*L*) required to store *s*_r_ given the soil properties

$$L\geq \frac{s_{r}}{\theta_{c}-\theta_{m}}$$ (2)

In the following sections, we calculate the daily required water storage (*s*_r,*i*_) using a simplified water balance approach with applied water and AET. Then, minimum required soil depth is calculated as the minimum required cover thickness using wet-extreme required water storage and soil parameters. All modeling was done in Python throughout unless otherwise noted, and all code is published in the repository for this paper (*41*).

#### Applied water calculation

ET cover thickness is dependent on the magnitude and timing of applied water events relative to atmospheric evaporative demand and plant transpiration. We identified that daily or finer temporal resolution is required to capture this, as coarser, time-averaged data can hide pulse events. Applied water describes when water reaches the soil column from rainfall or melt from frozen precipitation. Previous landfill literature identifies the need for accounting for snow temporally (*82*). We differentiate applied water from precipitation because precipitation commonly indicates when water falls from the atmosphere, but this can be different from when that water reaches the soil column for frozen forms of precipitation.

To calculate applied water, we acquired historic daily maximum temperature (*T*_max_, degrees Celsius), minimum temperature (*T*_min_, degrees Celsius), precipitation (*P*, millimeters), daylight average shortwave radiation (*R*_s_, watts per square meter), and daylength (*d*, seconds) from Daymet, version 4.1 (*78*). Daymet interpolates corrected weather station data from the Global Historical Climate Network Daily database with an algorithm trained for the US West (*83*). We acquired netCDF files of the requisite variables clipped to the US West states (Arizona, California, Colorado, Idaho, Montana, Nevada, New Mexico, Oregon, Utah, Washington, and Wyoming) for 2000–2021 using Pydaymet (*84*) in November 2024. The Daymet grid is a custom North American Lambert Conformal Conic (LCC) projection (hereafter, Daymet LCC) with a 1-km spatial resolution. We used this projection for all work presented here unless otherwise specified.

We calculated applied water for each pixel and day in the record. We partitioned precipitation into rainfall (*RF*, millimeters per day) and snowfall [*SF*, millimeters (liquid water equivalent) per day] using a temperature threshold. We calculated the daily average temperature (*T*_d_, degrees Celsius) by taking the average of *T*_max_ and *T*_min_. We considered using high temporal resolution temperature data to resolve a high-accuracy daily average temperature but found that the simple average of *T*_max_ and *T*_min_ was suitable for our use case, especially given the broad range of diurnal temperature cycles across our study area. *P* for a given day was classified as *RF* if *T*_d_ ≥ 0 and *SF* if not. *SF* for a given day (denoted with subscript *i*, starting with *i* = 0 following Python convention) is added to the snowpack (*SP*, millimeters) accumulated over time period of length *n* days whereby

$${\mathrm{SP}}_{i}={\mathrm{SF}}_{i}+\sum_{i=0}^{i-1}{\mathrm{SP}}_{n}$$ (3)

If *SP*_*i*_ > 0 and *T*_*d*,*i*_ > 0, we calculate daily snowmelt (*SM*) using a restricted degree-day radiation approach (*85*)

$${\mathrm{SM}}_{i}=a_{r}T_{d,i}+m_{Q}R_{n,i}$$ (4)

where *a*_*r*_ (millimeters per degrees Celsius) is a restricted degree-day factor, *T*_d_ is the average degree-day temperature above a base temperature (considered to be 0.0°C for this study), *m*_Q_ is a conversion factor from radiative energy flux density to snowmelt depth (2.6 mm m^2^ day^−1^ W^−1^) (*85*), and *R*_n_ is net radiation (watts per square meter). The restricted degree-day factor *a*_r_ ranges from 2.0 to 2.5 mm °C^−1^ (*86*) with lower values corresponding to days with lower wind speeds or lower humidity which reduce sensible heat transfer or increase latent heat dissipation respectively. We used an average *a*_r_ of 2.25 mm °C^−1^ following the sensitivity analysis described in the “Sensitivity to applied water snow parameterization” section. Because snow can be approximated as a blackbody for longwave radiation (*87*), *R*_n,*i*_ was calculated using *R*_s,*i*_ and *d*_i_ from Daymet as following

$$R_{n,i}=\left(1-\alpha \right)\left(\frac{d_{i}}{86400}\right)R_{s,i}$$ (5)

We define average snow albedo (α) as 0.74, an approximated average snow albedo between fresh snow and nearly ablated snow (*85*, *88*). Snow storage is calculated iteratively to account for daily changes. At the end of a time step, *SP* is initialized for the next day

$${\mathrm{SP}}_{i+1}={\mathrm{SP}}_{i}-{\mathrm{SM}}_{i}$$ (6)

Because we calculate these variables on an annual basis due to computational limits, snowpack from the last day of the prior year is used to initialize the following year, with the exception of the beginning of the record in 2000. Last, daily snowmelt and rainfall are summed to calculate applied water (*AW*, millimeters per day)

$${\mathrm{AW}}_{i}={\mathrm{SM}}_{i}+{\mathrm{RF}}_{i}$$ (7)

#### Geospatial historic water balance

We modified a simple one-layer “bucket” water balance model to quantify required water storage in the soil column. We assume that all *AW* enters the soil column and thus the reservoir and neglect any water removal mechanisms other than evapotranspiration, measured by AET. This approach eliminates the surface runoff term because surface runoff is highly heterogeneous and requires many parameters to be reasonably estimated, even without considering storage chamber engineering (*54*). We omitted runoff as the most conservative approach (i.e., resulting in higher predicted required water storage and a greater required cover thickness). We also neglect water balance factors that are small (e.g., sublimation and water vapor interactions with snowpack) or too challenging to quantify on a broad spatial scale (e.g., wind transport of snow). To enable calculation of maximum required water storage in the soil column to prevent percolation to biomass at depth, we specified the reservoir to be unconstrained (no “leaky bucket” simulation of base runoff or loss to groundwater). As such, any stored water is also available for evapotranspiration. We assume that no water enters the soil column laterally or from depth, which is supported by removing areas where bedrock depth or long-term modeled water table depth is less than 10 m (see the “Geospatial masking” section).

We retrieved daily AET rasters from the operational Simplified Surface Energy Balance (SSEBop) model in November 2024 for 2000–2021 (*79*, *89*). The SSEBop model processes Moderate Resolution Imaging Spectroradiometer satellite data with dynamic, pixel-specific boundary conditions to estimate the 8-day evapotranspiration fraction (*90*), which is then processed to a daily time step with daily reference evapotranspiration (*89*). We reprojected each daily AET GeoTIFF from WGS84 to Daymet LCC using cubic convolution to preserve extrema, clipped the reprojected rasters to the spatial extent of the study area, and compiled all rasters into a single netCDF along the time axis. We removed 31 December on leap years to match the Daymet convention.

Many pixels in the AET record indicate *AET*_*i*_ = 0 despite *s*_r,*i*_ > 0. Given the assumptions of our model, if water is present in the reservoir, it is available for loss via AET if the physical conditions permit. Annual cumulative potential evapotranspiration, the evapotranspiration that would occur over a year in an area with unlimited soil water, exceeds 750 mm for all areas in this region (*91*). Additionally, the SSEBop model uncertainty is highest in arid regions with low vegetation cover (as quantified by normalized difference vegetation index), high albedo, and high emissivity (*89*). When SSEBop was run with Landsat data across the US West, the median root mean square error was 0.50 mm day^−1^ (*92*). As such, we implemented an AET adjustment of 0.50 mm day^−1^ for individual days and pixels where (i) daily AET from SSEBop was 0 (*AET*_*i*_ = 0), (ii) the daily maximum temperature exceeded 0°C (*t*_max_ > 0), and (iii) there was no snowpack (*SP*_*i*_ = 0).

We iteratively calculated daily required water storage (*s*_r,*i*_, mm) for each pixel and *i*th day over 2000–2020 using the daily simplified water balance as the following, specifying that the storage reservoir cannot go below zero

$$s_{r,i}=\text{max}\left(0,\left\{ \begin{array}{c} i=0⟹{\mathrm{AW}}_{i}-{\mathrm{AET}}_{i}\\ i>0⟹s_{r,i-1}+{\mathrm{AW}}_{i}-{\mathrm{AET}}_{i} \end{array}\right.\right)$$ (8)

We considered 2000 to be an initialization year and removed it from the dataset after calculating the entire time series. The resulting daily required water storage record spans 2001 to 2020.

##### Sensitivity to applied water snow parameterization

To assess the sensitivity of required water to applied water snow parameterization, we conducted four calculations of *s*_r_ with different parameterization: (i) base case (*a*_r_ = 2.25 mm °C^−1^), (ii) low *a*_r_ (*a*_r, low_ = 2.50 mm °C^−1^), (iii) high *a*_r_ (*a*_r,high_ = 2.00 mm °C^−1^), and (iv) no snow, where precipitation *P* was used in place of *AW* in the water balance model described in the “Geospatial historic water balance” section. The results of this analysis are displayed in fig. S7. The value of *a*_r_ minimally affected results, with an average percent difference between *s*_r,95_ for the base case and low *a*_r_ (high *a*_r_) condition being 0.05% (−0.05%) across the US West.

#### Final minimum required soil cover thickness calculation

To translate from required water storage to burial depth, we use (i) the 95th percentile maximum daily required water storage (*s*_r,95_) to represent an extreme required water storage condition (given conservative assumptions about runoff) and (ii) available water content (*AWC*), equal to the difference between volumetric soil water content at field capacity and wilting point ($\mathrm{AWC}=\theta_{c}-\theta_{m}$) (see the “ET cover theory” section)*.* For *AWC* data, we retrieved the US Department of Agriculture gridded National Survey Geographic (gNATSGO) Database (*34*) in February 2024 and calculated depth-weighted average *AWC*, from the surface to the maximum sampled depth at a 30-m resolution for the entire region using R and ArcGIS Pro. gNATSGO is available with a native 30-m spatial resolution in Albers Equal Area Conic (NAD83), and we used cubic convolution to upscale and regrid the dataset to Daymet LCC with a 1-km resolution.

We assume that soil types and distributions have not and will not change on decadal scales (*93*). We calculated the minimum required soil cover thickness (*L*) for each pixel using a modified form of Eq. 2

$$L=\frac{S_{r,95}}{\mathrm{AWC}}$$ (9)

Regional- and state-level spatial statistics were conducted in Python, truncated at *L* = 10 m (Fig. 2).

#### Geospatial masking

We applied several raster masks to remove areas with conditions that either limited model validity or had constraints that would make implementation impossible. For continuous rasters, we used cubic convolution to regrid to the Daymet LCC projection at the original product resolution. For discrete rasters, we used nearest neighbor sampling to regrid to the Daymet LCC projection. We upscaled all raster masks and the *L* raster to 30-m resolution to match the highest resolution mask product before applying the mask. Before performing further analysis on the masked *L* layer, we downscaled it back to the 1-km resolution with nearest neighbor sampling.

We applied three raster masks: bedrock depth, water table depth, and land cover–type classifications. We removed areas where the bedrock was shallower than 10 m using the SoilGrids250m product (*94*). Not only does shallow bedrock prevents digging for biomass burial, but also shallow bedrock promotes a higher water table and more runoff that both render the water balance modeling assumptions about a free drainage bottom boundary condition and neglecting runoff less accurate. We removed areas where long-term average water table depth was modeled to be less than 10 m to reduce the likelihood of groundwater intrusion into the burial chamber and maintain a free drainage boundary condition (*95*, *96*). For land cover masking, we created a composite mask using the annual National Land Cover Database (NLCD) Collection 1 in December 2024, removing pixels classified as any of the following categories during the 2000–2020 study period: open water, perennial ice/snow, medium-intensity development, high-intensity development, pasture/hay, cultivated crops, woody wetlands, and emergent herbaceous wetlands (*97*). Open water, perennial ice/snow, and wetlands are mutually exclusive to biomass burial under ET soil covers because these regions are either under snow and ice or periodically or continually saturated with water. We removed all agricultural land classifications because irrigated agriculture would nullify the model results by adding an applied water source unaccounted for by the precipitation-based applied water model while amplifying the AET signal. We removed medium- and high-intensity developed pixels because most of the land surface area is covered with impervious surfaces, which would disrupt the model and reduce area available for biomass burial.

Last, we applied a vector mask to remove protected land areas using the Protected Areas Database of the United States 4 (PAD-US4) (*98*). We removed any land area with gap analysis project (GAP) status codes of 1 or 2. A GAP status code of 1 indicates “[a]n area having permanent protection from conversion of natural land cover and a mandated management plan in operation to maintain a natural state within which disturbance events (of natural type, frequency, intensity, and legacy) are permitted to proceed without interference or are mimicked through management” (*98*). A GAP status code 2 indicates “[a]n area having permanent protection from conversion of natural land cover and a mandated management plan in operation to maintain a primarily natural state, but which may receive uses or management practices that degrade the quality of existing natural communities, including suppression of natural disturbance” (*98*). All masks combined removed 172,061,750 ha or 57% of total land area.

### Life cycle assessment

The spatial distribution of minimum required soil cover thickness (*L*) enabled a geospatially explicit, attributional gate-to-grave LCA of biomass burial, tracing carbon from thinned residues through processing, transport, and ultimate fate over a 100-year period. The functional unit is one metric ton of CO_2_-equivalent (MTCO_2_e) initially contained in the harvest biomass. The output metric is carbon efficiency (η) representing the percentage of initial CO_2_e that remains stored after 100 years, net of any process, and decay emissions (*37*) and is calculated as follows

$$\eta =\frac{{\text{CO}}_{2}e_{i}-{\text{CO}}_{2}e_{p}-{\text{CO}}_{2}e_{d}}{{\text{CO}}_{2}e_{i}}\times 100$$ (10)

where ${\text{CO}}_{2}e_{i}$ is the MTCO_2_e contained in the initial thinned biomass, ${\text{CO}}_{2}e_{p}$ is the MTCO_2_e emissions resulting from all process emissions, and ${\text{CO}}_{2}e_{d}$ is the MTCO_2_e emissions resulting from decay. For all non-CO_2_ emissions, we use 100-year global warming potential (GWP_100_) conversions to assess CO_2_e contribution (see the “Pile burning” section). Most η values range from 0 to 100%. A negative η implies a carbon positive process (i.e., net emissions were greater than the total MTCO_2_e contained in the initial biomass) and could occur if process emissions are extreme or if emissions of high-GWP gases, such as CH_4_, offset the carbon stored. The η of biomass burial was compared with counterfactual fates of (i) natural decomposition and (ii) pile burning; and alternative end uses (ii) biochar production and on-site application (in situ biochar) and (iv) BECCS.

#### Counterfactual scenarios

To first assess the business-as-usual, or counterfactual fate of these residues, we model simplified scenarios of (i) natural decomposition and (ii) pile burning.

##### Natural decomposition

Biomass thinned and left on site will decay as described by

$$\frac{W_{t}}{W_{0}}=e^{-\mathrm{kt}}$$ (11)

where $\frac{W_{t}}{W_{0}}$ is the final proportion of the initial mass remaining on site after *t* time in years and *k* is the annual rate of decay. If material is left on site to decompose at an annual *k* rate of decay of 0.028 [softwood mean; (*99*)] over a time period of 100 years, then the final proportion of biomass remaining is 0.061 or 6.1%. If we assume that the initial and final percent carbon within the biomass is unchanged, then η of natural decomposition is 6.1%.

##### Pile burning

Biomass left on site to decompose is vulnerable to future wildfire. Therefore, forest managers use prescribed pile burning to remove these fuels from the landscape in the near term (*7*). As an alternative counterfactual fate, we assess emissions associated with pile burning using the fuel consumption and emissions software developed by the USFS, CONSUME model version 3.0 (*48*). Using the Pile Consumption Algorithm with CONSUME v3.0, we model combustion [use default 0.90 as proportion of mass consumed; (*48*)] distributed across flaming (70%), smoldering (15%), and residual (15%) stages to increase accuracy (*100*) of emission estimates. Fuel consumption across stages is then multiplied by phase-specific emission factors associated with each greenhouse gas or criteria air pollutant. We select emission factors derived for mixed conifer fuel types (*101*, *102*) for greenhouse gas emissions and the emission factors derived by Hardy (*103*) for particulate matter. Assuming a GWP_100_ for carbon monoxide (CO), CH_4_, and non-CH_4_ hydrocarbons to be 2, 28, and 8, respectively (*25*), we estimate the η of pile burning to be 4.5%.

#### Biomass burial carbon efficiency

As an alternative option to the counterfactual fates presented, we quantified the life cycle emissions associated with biomass burial postfelling to long-term storage (i.e., 100 years postburial). We elect to start postfelling, as the business-as-usual of forest management is to thin and then either scatter and decay or pile and burn. Given that the cutting of the tree happens in both cases, we begin our analysis after this point. Included in the analysis are emissions associated with forestry processes (delimbing, loading, unloading, and stacking green wood), transportation, process emissions from creating the storage chamber (excavating and repacking), emissions resulting from disturbance of soil carbon, and biomass decay and storage emissions over 100 years. We describe specific process steps for each model component below and present sources [table S1 and (*41*)].

##### Process metrics, forestry

To ensure accurate process emissions associated with ecological thinning in the US West, we report empirical data collected in the eastern Sierra Nevada (CA) by a collaborating startup company in their first year of operations conducting ecological thinning (*104*). For each machine involved in the operations, field crews recorded the volume of biomass handled per unit time and the corresponding diesel consumption. These data were used to calculate diesel use and associated CO_2_ emissions per short green ton of biomass processed [using a diesel carbon intensity of 0.0027 l MTCO_2_e per liter; (*105*)]. Values reported at this first site were typically substantially less efficient than empirical values reported in the literature for the same processes [e.g., (*106*, *107*)]. Given that we expect increased efficiency over time as operators gain experience, we assume a modest 19% increase in efficiency over time owing to operator improvement [based on measurements in (*106*) between experienced and inexperienced equipment operators]. For each process step, we take the mean of the measured value on site and the expected increase in efficiency to remain conservative and representative of the average operational efficiency. We follow the same procedure for calculating emissions resulting from delimbing (Pierce PMD-3348 Stroke-Boom Delimber; −0.0034 MTCO_2_e emitted per MTCO_2_e contained in initial biomass), stacking (Doosan DX225LL loader; −0.0016 MTCO_2_e per MTCO_2_e contained in initial biomass), and loading and unloading (Doosan DX225LL loader; −0.0016 MTCO_2_e per MTCO_2_e contained in initial biomass) (*104*). In all cases, we assume green biomass has a moisture content of 54% (*108*) and that 50% of the bone-dry biomass is carbon by weight (*109*).

##### Transportation

Transporting waste residues will be a key step in any forest biomass utilization approach, as it can be extremely costly, especially if the material is of low value (no end market to offset cost) or situated in particularly remote locations. We estimate transportation emissions based on a few key assumptions. First, we assume that the truck transport limit is 22.7 metric tons of biomass (*110*) and that all biomass is green when transported (50% moisture content). We use reported values from the literature for fuel efficiency in miles per gallon (mpg) of class 8 trucks on two road types, public highway (6.66 mpg) and forest roads (3.16 mpg) (*111*, *112*). We use unit conversions to convert from miles per gallon to liters per mile and, assuming that trucks are full of 22.7 metric tons of biomass, into MTCO_2_e emitted per MTCO_2_e in biomass per mile. We set the upper limit of transportation to a ∼161-km (100-mile) radius (Euclidean distance) around the biomass source. We justify this assumption because without a higher value end use for the biomass, it is unlikely to be economically viable to transport biomass farther than that upper bound distance if the sole revenues are from the voluntary carbon market. The ultimate route, including proportion of paved and unpaved roads, and associated emissions are selected by the network analysis in the “Geospatial LCA network analysis” section.

##### Process metrics, storage chamber

At the burial site, the storage chamber will be excavated, filled with waste biomass, and then repacked with a bulldozer. To estimate emissions associated with the process of creating the storage chamber, we first model emissions resulting from excavation of soil based on field-measured values of excavator throughput (empirical field data report 60 to 80 yard^3^ hour^−1^; mean, 70 yard^3^ hour^−1^) (*104*) and excavator diesel use (43.7 to 55.0 liters hour^−1^; mean, 49.4 liters hour^−1^) (*113*) and convert these values to liters of diesel burned per cubic meter excavated.

To then estimate the liters of diesel burned per MTCO_2_e in initial biomass, we assume that, for every 1 m^2^ of storage chamber surface area, the storage chamber volume under the soil cover is 3 m^3^ and packed with an efficiency of 50%. Last, to ensure sufficient excavation to accommodate the volume required for the incoming biomass, we assume that the amount of excavation required is 1.5 times the soil cover thickness (*L*) for every 1 m^2^ of storage chamber surface area. For the repacking of the storage chamber, we use the same process, based on field measured values of 1.6 liters of diesel burned per cubic meters of earth moved (*104*) and assume the same volume of earth moved as above. Last, for both excavating and bulldozing, we multiply the liters burned per MTCO_2_e in biomass by the diesel carbon intensity (*105*) of the process to derive the MTCO_2_e emitted per MTCO_2_e in biomass.

##### Soil carbon

Because soil carbon varies across the US West, we performed soil carbon calculations geospatially. The first layer used is the modeled *L* raster described in the “Minimum soil cover thickness (*L*) modeling” section. Using the same methods described for acquiring gNATSGO *AWC* in the “Final minimum required soil cover thickness calculation” section, we generated a raster of organic matter (*34*). We acquired a depth-averaged soil bulk density raster product calculated from gNATSGO data (*35*). For both organic matter and soil bulk density rasters, we upscaled and reprojected the data with cubic convolution to 1-km Daymet LCC to match the projection and grid of *L* in QGIS 3.34.15-Prizren. We calculate all following for each pixel.

To estimate the emissions from soil organic carbon pools, we first calculate the volume of soil that is disturbed and exposed to oxygen through the excavation process per unit MTCO_2_e contained in initial biomass. This volume, calculated in the “Process metrics, storage chamber” section, is a function of burial depth, as quantified by the modeled *L*. To next estimate the amount of carbon that is contained in the aforementioned volume of soil, we then take the soil bulk density value and convert it to soil percent carbon via the van Bemmelen factor (divide by 1.724) (*114*). Given the soil bulk density, we then calculate the total amount of MTCO_2_e contained in the volume of soil disturbed per 1 MTCO_2_e in biomass. For a final step to estimate emissions, we assume that this one-time disturbance results in oxidation of 10% of the soil carbon. We suggest that this value is on the conservative side, given modest oxidation rates observed in studies that implemented annual disturbances over a 30-year period, especially in soils that started with low carbon content (*115*), and we test LCA sensitivity to this parameterization in the “Biomass burial LCA sensitivity analysis” section.

##### Storage chamber emissions

Once the storage chamber is refilled and compacted by the bulldozer, we model 100 years of emissions of the wood contained in the storage chamber. To estimate the proportion of the biomass that is vulnerable to decay, we first reviewed the available literature measuring biochemical methane potential and the fraction of degradable organic carbon vulnerable to decay (DOC_f_) (*26*, *28*). DOC_f_ is a key factor used in the landfill literature to estimate the amount of CH_4_ that a given feedstock will produce in an anaerobic setting. While other biomass burial LCAs use much lower DOC_f_ values for softwoods (e.g., 0.0347) (*42*), we select the Intergovernmental Panel on Climate Change average for wood waste, 0.088 (*26*) (but test the lower DOC_f_ later in the “Biomass burial LCA sensitivity analysis” section). We assume that the complete DOC_f_ will degrade over 100 years and that this decomposition will be into 50% CO_2_ and 50% CH_4_ on a molar basis (*26*). Last, given that ET covers have been shown to have relatively high oxidation of CH_4_ to CO_2_ in covers (>60%) (*116*) and that oxidation rates up to 100% have been observed and modeled for scenarios involving low concentrations of CH_4_ (*33*), which will likely be representative of a burial of softwood boles, we elect to use an initial oxidation rate of 0.75. We test the sensitivity of the LCA to this parameterization in the “Biomass burial LCA sensitivity analysis” section. The final emissions assume that any CH_4_ oxidized in the storage chamber cover will be emitted as CO_2_. For the CH_4_ that is emitted, we use a GWP_100_ of 28 to convert to MTCO_2_e and add this value to the emissions of CO_2_ expected from DOC_f_ decay for a final estimate of storage chamber emissions over 100 years.

##### Geospatial model components

Process emissions from forestry (−0.0066 MTCO_2_e per MTCO_2_e in biomass) and storage chamber emissions (−0.1889 MTCO_2_e per MTCO_2_e in biomass) are independent of their local context. However, other variables are informed by underlying geospatial variables: Soil carbon is dependent on soil organic matter, bulk density, and burial depth. Process emissions from creating the storage chamber are dependent the amount of earth that needs to be moved, which is a function of burial depth and, therefore, *L*. Last, transportation emissions are dependent on the distance traveled on each road type, which is selected via the network analysis in the “Geospatial LCA network analysis” section.

#### Carbon efficiency of alternative BiCRS approaches

To understand and compare the carbon efficiency of burial (*η*_b_) with other potential higher or better use BiCRS pathways, we compiled a realistic, near-term estimate of carbon efficiency for (i) biochar via slow pyrolysis at 350/450°C with on-site application, η_c_; and (ii) a bioelectricity BECCS pathway, η_B_. Both alternative end uses are based on Chiquier *et al.* (*37*).

We elect to use the slow pyrolysis pathway that uses forestry residues to generate biochar and apply them in forests as it is the most representative of our scenario. In this scenario, η_c_ is 43.5% and includes emissions from chipping biomass on site (decreases η_c_ by 1.7%), slow pyrolysis at 350° to 450°C (decreases η_c_ by 39.9%), biochar application (decreases η_c_ by 0.2%), and emissions from nonstable biochar over 100 years (decreases η_c_ by 14.1%) [see the supplementary materials of (*37*) for full model details; any biomass and biochar transportation emissions are excluded to maximize potential η_c_ relative to η_b_].

We model our BECCS pathway after the forestry residues derived from marginal lands scenario from (*37*). To enable direct comparison, we combine our LCA calculations of forestry and transportation emissions (delimbing, loading, transporting, unloading, and stacking raw biomass) with those reported for biomass processing (shredding and drying) and downstream BECCS emissions (carbon capture and storage, inclusive of CO_2_ transport) in (*37*) for a final η_B_ estimate. For the biomass transportation emissions, we use the same assumptions as for the transportation module in the biomass burial LCA. Therefore, η_B_ is a function of the distance traveled on each road type. Last, we set the maximum spatial radius around BECCS facilities to ∼402 km (250 miles), as the products of these processes are of far higher value and may justify more costs and emissions from the forest to the facility location [see (*41*) for full model details].

#### Geospatial LCA network analysis

As described above, biomass burial and BECCS LCAs are implemented geospatially because they require transportation of the forest residues offsite to an end location, and the biomass burial LCA is a function of spatially variable soil data. To implement the geospatial component of the LCA, we first acquired the road network for the entire US West in April 2025 using OSMnx 2.0.4 in Python to access OpenStreetMap (*36*). We selected any potentially drivable road: any highway including motorway, trunk, primary, secondary, tertiary, residential, unclassified, service, track, and road classifications. The resulting network (MultiDiGraph) contains millions of directional roads (edges) and intersections (nodes) with additional information about road types, conditions, and driving direction. By default, OSMnx acquires the networks under WGS84, but distance-based analysis needs to be performed with a projected CRS, so we projected the network to Daymet LCC with units of kilometers. We performed all mile-to-kilometer unit conversions throughout as necessary.

We weighted each road as a function of edge length and carbon emissions factor determined by road type, where unpaved roads were assigned the forest road factor and paved roads were assigned the public highway factor described in the “Transportation” section (*111*, *112*). We classified unpaved roads as any tracks, or service or unclassified highways with one of the following road surface tags: unpaved, compacted, fine gravel, gravel, rock, pebblestone, ground, dirt, or earth. For any roads that did not fall under these categories, we used the public highway factor.

To estimate locations and quantities of nonmerchantable biomass residues, we used a model generated by Pett-Ridge *et al.* (*6*) that estimates county-level residue generation under the USFS 10-Year Wildfire Crisis Strategy Plan (*5*). The model assumes conservative thinning prescriptions (i.e., thinning from below to 80% full stocking), biomass size limits [<30.5-cm (12-inch) diameter], and access constraints [e.g., slope of >40% or >804.7 m (0.5 miles) from roads excluded] and includes counties in order of decreasing wildfire hazard potential until treatment targets are met. The model results in total acreage and bone-dry short tons of biomass (converted to bone-dry metric tons) to be treated for each of 173 counties by accessibility classification (figs. S1 and S2). Accessibility classifications are all possible combinations of distance to road [0 to 152.4 m (0 to 500 feet), 152.4 to 304.8 m (500 to 1000 feet), and 304.8 to 804.7 m (1000 feet to 0.5 miles)] and slope (<20%, 20 to 40%). For counties modeled to have any nonzero quantity of residues for removal, we assigned the county centroid as a start node in the network and connected it to the network with a bidirectional edge following the shortest Euclidean distance from the new node to an existing node and weighted with the forestry road factor as a function of edge length.

For the biomass burial LCA network creation, we considered any pixel as a potential end node that (i) had *L* < 6.67 m (thereby allowing any *L* up to 5 m plus additional modeled excavation depth) and (ii) was less than 804.7 m (0.5 miles, Euclidean distance) from any part of the acquired road network. We created an 804.7-m (0.5-mile) buffer around the road network, mirroring the access constraints outlined in the biomass availability modeling and used this to clip the raster resulting from the soil carbon calculations described in the “Soil carbon” section. For each of the 173 county centroid start nodes, we created individual networks by clipping the network for the entire US West to a ∼161-km (100-mile) radius surrounding the start node. For each burial end node within each 100-mile radius, we connected the burial end node to the existing network with a bidirectional edge from the new node to the closest existing node as measured by Euclidean distance and weighted the new edge with the forestry road factor as a function of edge length plus the cumulative biomass burial carbon efficiency from the LCA excluding transportation.

For the BECCS LCA network creation, we used a series of modeled BECCS facility locations derived from the BILT model published in (*6*) as BECCS end nodes. The BECCS facilities are modeled using the percentage of biomass removed relative to thinning commitments as a proxy for time, with 25, 50, 75, 90, and 99% biomass removal scenarios. For our analysis, we included any facility in the US West that was modeled to run any type of forestry residue (excluding pulp and paper). For each of the 173 county centroid start nodes, we created individual networks by clipping the network for the entire US West to a ∼402-km (250-mile) radius surrounding the start node. We created a copy of each of these networks for each of the biomass removal scenarios if BECCS facilities were modeled to exist within the 250-mile radius for that county within a given biomass removal scenario. For each BECCS end node within the 250-mile radius, we connected the BECCS end node to the existing network with a bidirectional edge following the shortest Euclidean distance from the new node to an existing node and weighted it with the highway road factor as a function of edge length plus the BECCS carbon efficiency from the LCA excluding transportation.

For compatibility with the network analysis algorithm, we assigned each edge within each network to a weight of 1 minus the carbon efficiency (expressed as a proportion; 1 − η) of that edge. We used the single-source Dijkstra algorithm from NetworkX to calculate the lowest cumulative weighted path from each county centroid start node to available end nodes (*117*, *118*). For the optimal path, the algorithm returns the cumulative 1 − η, path length, and path shapefile. We converted 1 − η back to carbon efficiency (η) for the remainder of the analysis. To calculate the η associated with two-way travel, we doubled transport-specific η and added it to the end node η.

We performed this minimization for every network in the burial only scenario and each BECCS scenario. For each BECCS scenario, we compared the biomass burial and BECCS outcomes for each county and selected the path with the lowest 1 − η to select the most favorable outcome across pathways. We also compared all of the LCA outcomes to the in situ and counterfactual fates and found that both biomass burial and BECCS outcomes exceeded the carbon efficiencies of on-site natural decomposition, piling and burning, and biochar production with in situ application.

For the biomass burial only and each of the BECCS biomass removal scenarios, we calculated descriptive statistics (mean, median, SD, and range) of the types of outcomes, cumulative carbon efficiencies, and transportation distances. We also analyzed the magnitude of the thresholds between biomass burial and BECCS outcomes when both outcomes were possible under a given biomass removal scenario.

##### Biomass burial LCA sensitivity analysis

To assess the importance of specific LCA parameterization and highlight critical factors for biomass burial implementations, we conducted a spatial sensitivity analysis on the pretransportation biomass burial LCA. For this analysis, we varied DOC_f_, fraction of CH_4_ oxidation in the cover, and fraction of soil carbon oxidized during excavation one at a time and calculated the resulting carbon efficiencies excluding transportation for every burial end node included in the LCA. The spatially averaged results are displayed in Fig. 4 with ±1 SD shading. This analysis illustrated that, within our LCA, the highest carbon efficiency outcomes corresponded to the lowest modeled DOC_f_ (DOC_f_ = 0.03), and the lowest carbon efficiency outcomes corresponded to the lowest modeled fraction of CH_4_ oxidized in the soil cover (0.10). Therefore, we conducted all analysis outlined in the “Geospatial LCA network analysis” section for the two scenarios where (i) DOC_f_ equals 0.030 and (ii) the fraction of CH_4_ oxidized in the cover equals 0.10, and the results are plotted, in the same manner as Fig. 3, in figs. S3 and S4, respectively. Because mean *L* of end nodes selected by the base-case network analysis was less than the ∼1- to 2-m or greater depth often required to reach the depth of oxygen extinction (*38*) to maintain anoxic conditions, we conducted an additional network analysis with a minimum *L* of 1 m (if any pixel had an *L* < 1 m, it was set to 1 m for LCA calculations) and report the results in the text.

### Technoeconomic analysis

To understand the relative importance and effects of economic factors for each of the pathways analyzed, we conducted a simplified, comparative TEA using the results of our LCA study and published data. All costs are normalized to a functional unit of 2026 United States dollar per MTCO_2_e in the initial biomass ($ MTCO_2_e_i_^−1^). For the counterfactual (pile burning) and each BiCRS approach (biochar, burial, and BECCS), we created a cost function. We use the following generic equation for each approach

y=−φ−αx+η(ρ+ξ−κ−ω) (12)

where *y* is net income ($ MTCO_2_e_i_^−1^), φ is the cost of forest processes (thinning and skidding) per MTCO_2_e in initial biomass ($ MTCO_2_e_i_^−1^), α is cost of roundtrip transportation per one-way distance per MTCO_2_e in initial biomass ($ MTCO_2_e_i_^−1^ km^−1^), and *x* is one-way road transportation distance (kilometers). The term η represents carbon efficiency as a fraction, MTCO_2_e_s_ MTCO_2_e_i_^−1^, where MTCO_2_e_s_ is the MTCO_2_e stored after 100 years, inclusive of all process emissions (1 MTCO_2_e_s_ = 1 carbon credit). The carbon credit price is represented by ρ ($ MTCO_2_e_s_^−1^), and the revenue from coproducts, normalized per carbon credit produced, is ξ ($ MTCO_2_e_s_^−1^). κ and ω represent CapEx and OpEx, respectively, per carbon credit ($ MTCO_2_e_s_^−1^).

We use the following simplifying assumptions: (i) mass loss is limited to biogenic carbon losses (e.g., biomass decay and process emissions); (ii) costs are treated as time-invariant, except where discounting is explicitly applied; (iii) the effect of transportation distance on carbon efficiency is minimal and thus not explicitly modeled; and (iv) bone-dry biomass is 50% carbon by mass (*109*). This analysis does not place valuation on the difference between the durability and risk of reversal of storage between these pathways beyond any impact on the carbon efficiency modeled for the 100-year time horizon of the LCA. Details for each calculation are described below, with additional code and spreadsheets stored at (*41*).

For each parameter in Eq. 12, we identify a base-case value representing the most likely or literature-supported estimate, along with low and high bounds to capture uncertainty and different scenarios. These values are presented in table S2. Parameter ranges of φ, α, and ρ are common across all pathways involving forest processes (all pathways), biomass transportation (burial and BECCS), and carbon credit generation (burial, BECCS, and biochar), respectively.

We calculate φ by summing the cost of felling, skidding, and piling biomass at a landing where it could then be used for any of the pathways. Literature reports felling costs of $10.13 per bone-dry short ton (BDT), skidding costs of $9.67 BDT^−1^ plus $0.92 BDT^−1^ per 100 feet of skidding, and piling costs of $6.49 BDT^−1^ (*119*). We apply these costs to six accessibility classes of residual biomass modeled from Pett-Ridge *et al.* (*6*) (see the “Geospatial LCA network analysis” section), which encompass all combinations of distance to roads [0 to 152.4 m (0 to 500 feet), 152.4 to 304.8 m (500 to 1000 feet), and 304.8 to 804.7 m (1000 feet to 0.5 miles)] and slope (<20%, 20 to 40%). To account for the variation of skidding distance within the accessibility classifications, we first calculate the total cost for each 100-feet increment of skidding distance and then take the average of the distances included in a given accessibility class range. Because forestry costs increase on steep slopes, we use a steepness multiplier of 1.47 for accessibility classes with slope 20 to 40% (*120*). We convert from short tons to metric tons of biomass and from metric tons of biomass to the functional unit of $ MTCO_2_e_i_^−1^. We set the low bound cost as the cost of the most accessible classification, 0 to 500 feet from the road and slope of <0% ($17.17 MTCO_2_e_i_^−1^), the high bound cost as the cost of the least accessible classification, 1000 feet to 0.5 miles from the road and slope of 20 to 40% ($38.03 MTCO_2_e_i_^−1^), and the base cost as the average across all six classifications ($25.93 MTCO_2_e_i_^−1^).

We calculate α using reported values of cost per ton of biomass per distance traveled and assume fully loaded logging trucks with a wood moisture content of 54% (*44*). For the carbon credit price ρ, we adopt a range of $50 to 300 MTCO_2_e_s_^−1^: the low bound of $50 MTCO_2_e_s_^−1^ representing aspirational affordability targets for large-scale deployment; a base value of $100 MTCO_2_e_s_^−1^ now consistent with emerging estimates for lower-cost CDR pathways such as burial; and an upper bound of $300 MTCO_2_e_s_^−1^ aligned with higher-cost, engineered CDR approaches (*6*). Values of *x*, η, κ, ω, and ξ are pathway specific and described further below.

#### Pile burning

We defined the cost equation for pile burning as follows

$$y_{p}=-\varphi -\omega_{p}$$ (13)

where the subscript “p” indicates pile burning and ω_p_ is in units of $ MTCO_2_e_i_^−1^. We assume that there is no road transportation of biomass (*x*_p_ = 0), the operators own all required equipment to conduct pile burns (κ_p_ = 0), no carbon credits are generated (ρ_p_ = 0), and no salable products are generated (ξ_p_ = 0).

As all felling, skidding, and piling is included in φ, pile burning OpEx, ω_p_, consists only of the cost of burning the piles. To estimate the lower bound costs of pile burning, we used the lowest reported value for burning in the published literature [$213 ha^−1^; (*121*)], which is consistent with lower bound estimates reported elsewhere from the USFS Activity Tracking System database [$252 ha^−1^; *n* = 3337; (*10*)]. Given conservative reports of 11.2 BDMT ha^−1^ in nonmerchantable residues (*121*), the lower bound of burning residues is $10.38 MTCO_2_e_i_^−1^.

For locations suitable for machine piles, Barker *et al.* (*10*) report costs ranging from $399 ha^−1^ for burning machine piles to $726 ha^−1^ for burning hand piles. In this study, the mean residue generation was 33.3 BDMT ha^−1^. The resulting costs of piling and burning residues from wildfire mitigation thinning activities are $22.91 MTCO2e_i_^−1^ for machine piles, taken to be the base cost, and $41.69 MTCO2e_i_^−1^, taken to be the upper bound cost.

#### Biochar

We defined the cost equation for biochar production as

$$y_{c}=-\varphi +\eta_{c}\left(\rho +\xi_{c}-\kappa_{c}-\omega_{c}\right)$$ (14)

where the subscript “c” indicates biochar production. We assume in situ production of the biochar such that there is no road transportation of biomass (*x*_c_ = 0). Biochar carbon efficiency, η_c_, varies widely based on initial feedstock and thermochemical conversion; we use the value calculated in this LCA as the base case (0.435) and include a representative range from the literature [0.16 to 0.459; (*37*, *122*)].

To match LCA assumptions of in situ biochar, we model costs of a mobile biochar unit deployed in forest settings, capable of producing 1350 metric tons (MT) of biochar per year (80% carbon content) (*123*). Biochar CapEx (κ_c_) and OpEx (ω_c_) are estimated to be $781,500 (one time cost, hereafter κ_c,total_) and $93.78 MTCO_2_e_s_^−1^, respectively (*123*). We annualize κ_c,total_ using the following equation

$$\kappa_{c,\text{annual}}=\kappa_{c,\text{total}}\times \frac{r\;{\left(1+r\right)}^{n}}{{\left(1+r\right)}^{n}-1}$$ (15)

where *r* is the discount rate (0.10) and *n* is the project lifetime in years (20 years). Using these parameters, κ_c,annual_ is $91,435.50 year^−1^. Assuming annual biochar production from one mobile unit (1350 metric tons year^−1^) and the number of carbon credits per ton of biochar produced (2.5 credits) (*123*), we estimate that κ_c_ is $27.09 MTCO2e_s_^−1^. For the parameter space of κ_c_ and ω_c_, we use a theoretical range of ±$10 MTCO_2_e_s_^−1^ and ± $20 MTCO_2_e_s_^−1^, respectively.

While in situ biochar production implies that much of the biochar produced will remain on site, production and sale of biochar as an agricultural amendment may meaningfully affect project economics. Therefore, we assess a range of potential product prices for biochar produced, ξ_c_, with a lower bound of $0, a base case of $83.36 MTCO_2_e_s_^−1^ [biochar product price of $208 metric tons^−1^; (*123*)], and an upper bound of $560.00 MTCO_2_e_s_^−1^ [biochar product price of $1400 metric tons^−1^; (*124*)], although transportation costs for these end uses are excluded.

#### Biomass burial

We defined the cost equation for biomass burial as

$$y_{b}=-\varphi -\alpha x_{b}+\eta_{b}\left(\rho -\kappa_{b}-\omega_{b}\right)$$ (16)

where the subscript “b” indicates biomass burial. Because burial generates no salable products, ξ_b_ = 0. The values for *x*_b_ are derived from the results of the network-based LCA, where the low bound equals the shortest modeled one-way transportation distance to burial (8 km), the base case equals the mean distance (140 km), and the upper bound equals the highest distance (295 km). We also use values for η_b_ from this analysis (as fractions), where the average η_b_ of all burial outcomes is the base case (0.783, Fig. 3A), the low bound is equal to the mean η_b_ from the low CH_4_ cover oxidation sensitivity analysis (Fig. 4) minus 0.01 for approximated transportation emissions (0.511), and the upper bound is equal to the mean η_b_ from the low DOC_f_ sensitivity analysis (Fig. 4) minus 0.01 for approximated transportation emissions (0.897). We identify ranges for κ_b_ and ω_b_ from TEAs in literature (table S2) (*16*, *19*).

#### Bioenergy with carbon capture and storage

We defined the cost equation for BECCS as

$$y_{B}=-\varphi -{\alpha x}_{B}+\eta_{B}\left(\rho +\xi_{B}-\kappa_{B}-\omega_{B}\right)$$ (17)

where the subscript “B” indicates BECCS. The values for *x*_B_ are derived from the results of the network-based LCA, where the low bound equals the shortest modeled one-way transportation distance to BECCS (14 km), the base case equals the mean distance (196 km), and the upper bound equals the highest distance (798 km). Values for η_B_ are similarly derived, where the low bound is the lowest carbon efficiency modeled (0.767) and the base case is the mean (0.809), but the upper bound is selected from literature (0.878) (*14*).

Values for κ_B_, ω_B_, and ξ_B_ are predominantly calculated from the Pett-Ridge *et al.* (*6*) modeling used for the BECCS end nodes in the LCA. These data are shared in the Zenodo archive for this project (*41*). Four types of BECCS facilities are included: combustion to electricity with carbon capture and storage (CCS), pyrolysis to asphalt with CCS, gasification to H_2_ with CCS, and sawmill with combustion of waste with CCS. For each modeled BECCS facility, κ_B_ was calculated as the reported capital cost divided by the tons of CO_2_ stored. We calculated ω_B_ for each facility by summing the reported operating cost, production cost, CO_2_ transportation cost, and CO_2_ injection cost and dividing this value by the tons of CO_2_ stored. We calculated ξ_B_ by dividing revenue by the tons of CO_2_ stored. For each variable, we used the minimum, mean, and maximum for the low, base, and upper bounds, respectively (table S2). The one exception is the upper bound of ξ_B_. Pett-Ridge *et al.* (*6*) stated that assumptions of product revenue are “conservative,” assuming “only historical averages of fossil-based product prices with no incentives.” We approximated a green hydrogen tax incentive of $3 kg H_2_^−1^ (*43*), where 100 kg H_2_ are produced per bone-dry ton of initial biomass (*125*). We added this incentive revenue ($216.64 MTCO_2_e_s_^−1^) to the highest ξ_B_ for the modeled gasification facilities ($154.98 MTCO_2_e_s_^−1^) to set the upper bound ξ_B_ ($345.69 MTCO_2_e_s_^−1^), and we modeled an additional base-case where this tax incentive revenue was added to base ξ_B_ (ξ_B_ = $318.73 MTCO_2_e_s_^−1^).

#### Comparative analysis

For each pathway, we calculated net income for three scenarios: (i) base case, where all of the base values are used; (ii) highest profit case, where all of the most financially favorable end range values are used; and (iii) highest cost case, where all of the least financially favorable end range values are used. We also performed a sensitivity analysis of all parameters across the parameter space for each pathway (fig. S5). For each parameter, we divided the parameter space into 10, calculated the percent difference in this value from the base value, and calculated net income associated with this varied parameter and the base value of all other parameters.

### Wildfire mitigation impact analysis

To understand the potential opportunities represented by the LCA and TEA results, we integrated these models with the nonmerchantable biomass availability described in the “Geospatial LCA network analysis” section to calculate the net cost of treating forests in the US West. For all modeled nonmerchantable biomass in each of the six accessibility classifications and counties, we calculate (i) forestry costs of thinning, (ii) pile burning costs, (iii) burial costs, (iv) BECCS costs, and (v) biochar costs from the initial bone-dry biomass modeled to be thinned in wildfire mitigation. We used the TEA equations described in the “Technoeconomic analysis” section with county-specific LCA results from the burial only and 90% removal with BECCS scenarios for transportation distance (*x*) and carbon efficiency (η). For all other parameters, we assumed the base-case values. We focused our analysis on the most accessible acreage (0 to 500 feet from roads, <20% slope) (fig. S2), but all results are available in (*41*).

The US Wildland Fire Service, the consolidated federal organization bringing together wildland fire resources from USFS and the Department of Interior, requested a 2026 budget of $770,050,000 for hazardous fuel management activities on 3.5 million ac of lands (*46*). We calculated the five total costs above for treating the most accessible acreage by county by decreasing wildfire hazard potential until 3.5 million ac was reached. This resulted in the most accessible acreage being treated completely in 56 counties, with one additional county being ∼20% treated (fig. S6), and the generation of 43 million BDMT of residues.

To next assess the criteria air pollutant (PM_2.5_) emissions associated with pile burning this material (in addition to MTCO_2_e emissions, see the “Pile burning” section), we used the Pile Consumption Algorithm within the CONSUME model version 3.0 (*48*) and assumptions for pile size, volume, consumable mass of wood (90%), and consumption across combustion phases from the “Pile burning” section. Particulate matter (PM_2.5_) emissions were estimated for the total consumed mass of wood using emission factors derived from Hardy (*103*) (clean piles, 13.5 pounds per short ton). Last, to estimate PM_2.5_ emissions from in situ biochar produced in an air curtain burner, we used an emission factor of 1.1 pounds per short ton [see (*126*) and citations within] and assume complete consumption.

## Supplementary Materials

### This PDF file includes:

Provenance Statement Figs. S1 to S7 Tables S1 and S2 References
