# Supplementary material for: Near-term, geospatial opportunity for biomass carbon storage to address the wildfire and climate crises
Source: Sci Adv. 2026 Aug 26;12(35):eaee6185. doi: 10.1126/sciadv.aee6185 (PMC13510600; doi:10.1126/sciadv.aee6185)
Supplement: Supplementary file 1 [file sciadv.aee6185_sm.pdf]

Science Advances
AAAS

Supplementary Materials for

Near-term, geospatial opportunity for biomass carbon storage to address the wildfire and climate crises

Leah K. Clayton *et al.*

Corresponding author: Leah K. Clayton, leahkc@stanford.edu; Sinéad M. Crotty, sinead.crotty@cclab.org

Sci. Adv. **12**, eaee6185 (2026)
DOI: 10.1126/sciadv.aee6185

This PDF file includes:

Provenance Statement
Figs. S1 to S7
Tables S1 and S2
References

Provenance Statement: No fossils, artifacts, ecofacts, and/or human remains were collected or used as a part of this study.

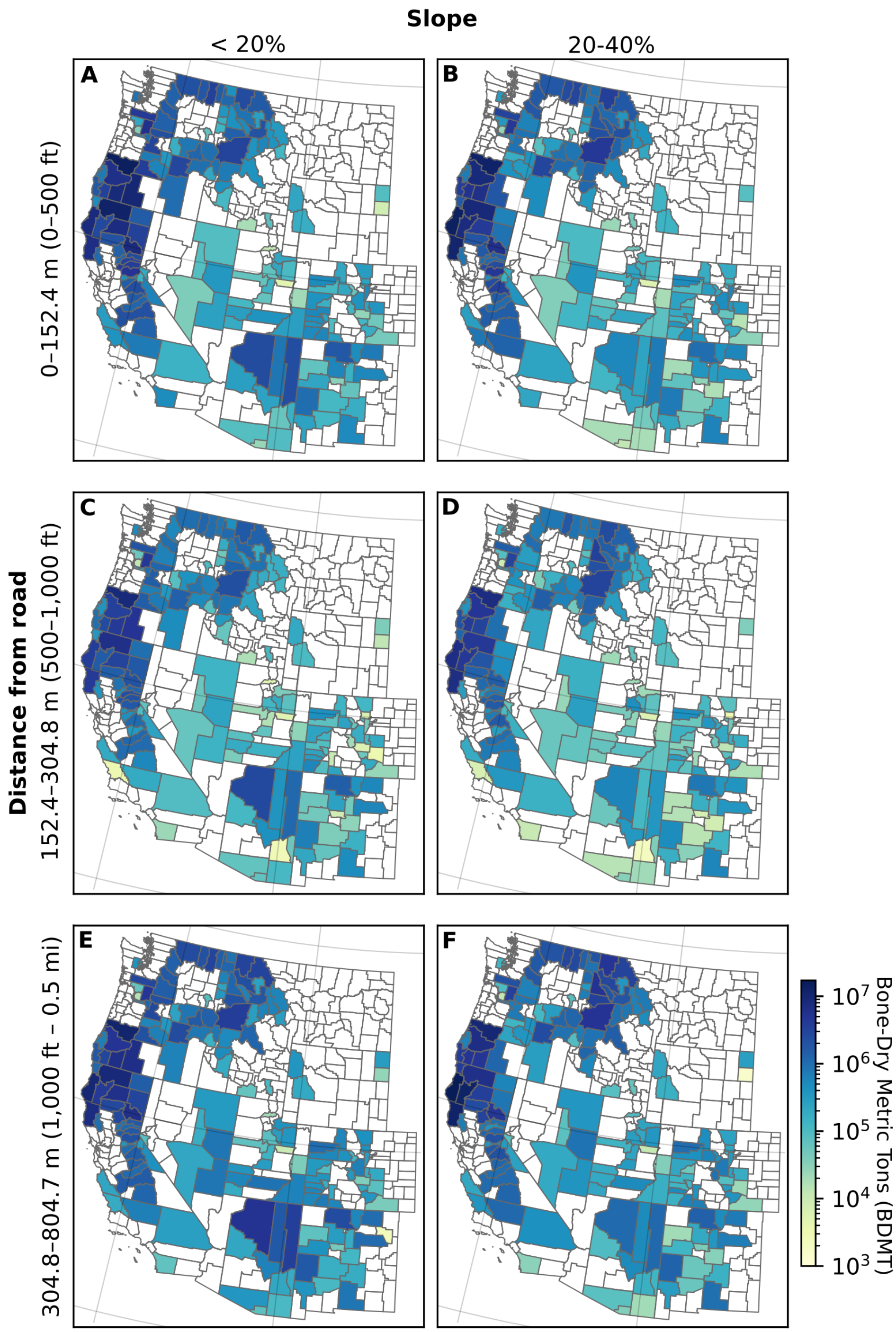

Fig. S1. Modeled non-merchantable, residual biomass availability in the western United States. Bone-dry metric tons (BDMT) of non-merchantable biomass modeled to be produced by thinning ~28 million hectares (70 million acres) in the highest wildfire risk counties by 2032 (*5*, *6*). Note: counties in white likely have non-merchantable biomass, but they were not included in the model which selected regions in order of cascading wildfire hazard potential. The panels correspond to each accessibility classification as a pair of distance from road and slope classification. For distances from the road of 0–152.4 m (0–500 ft), **A** and **B** correspond to slope <20% and 20–40%, respectively. For 152.4–304.8 m (500–1,000 ft), **C** and **D** correspond to slope <20% and 20–40%, respectively. For 304.8–804.7 m (1,000 ft – 0.5 mi), **E** and **F** correspond to slope <20% and 20-40%, respectively.

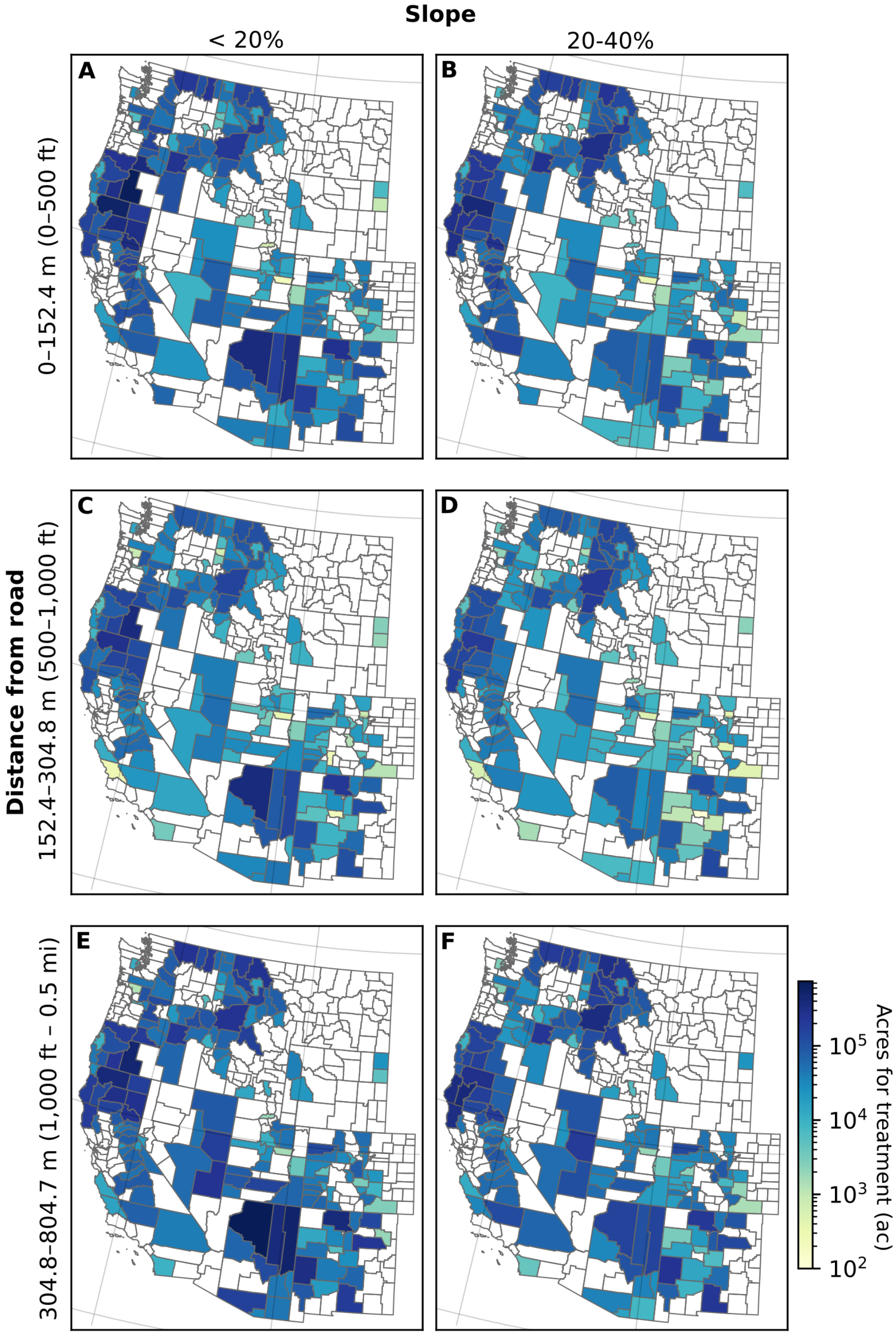

Fig. S2. Modeled acreage to be treated for wildfire risk reduction in the western United States. County acreage allocation of thinning ~28 million hectares (70 million acres) in the highest wildfire risk counties by 2032 by accessibility classification (*5*, *6*). Note: counties in white likely have acreage to be treated for wildfire risk reduction, but they were not included in the model which selected regions in order of cascading wildfire hazard potential. The panels correspond to each accessibility classification as a pair of distance from road and slope classification. For distances from the road of 0–152.4 m (0–500 ft), **A** and **B** correspond to slope <20% and 20–40%, respectively. For 152.4–304.8 m (500–1,000 ft), **C** and **D** correspond to slope <20% and 20–40%, respectively. For 304.8–804.7 m (1,000 ft – 0.5 mi), **E** and **F** correspond to slope <20% and 20-40%, respectively.

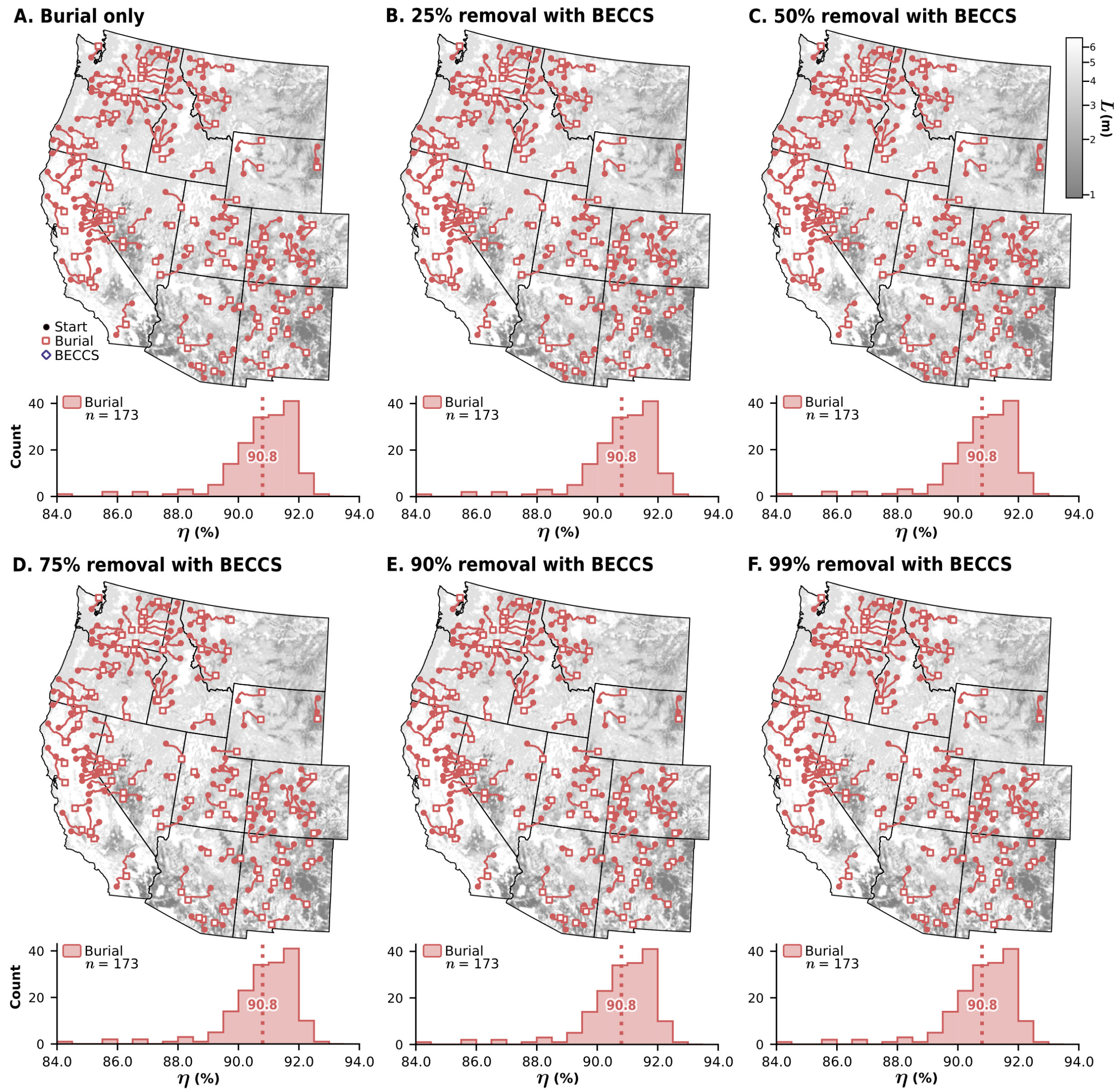

Fig. S3. Optimized modeled carbon efficiency (η) pathways for biomass burial and bioenergy with carbon capture and storage (BECCS) over six development scenarios for the low degradable fraction of organic carbon ($DOC_f = 0.03$) sensitivity analysis. Each panel displays (1) a map of modeled minimum soil cover thickness (***L***) overlain with the network routes between a biomass start node (solid circle) and the most carbon-efficient end node (square for burial, diamond for BECCS) for each county with thinned biomass; and (2) a histogram of $\boldsymbol{\eta}$, classified by outcome, with the labeled dotted line indicating the mean for each classification. Pathways that are optimized by burial fates are in red. No pathways were optimized with BECCS fates. Each panel represents a different BECCS scenario, where **A** represents a burial-only scenario with no BECCS infrastructure, and **B-F** illustrate BECCS scenarios (*6*) where increasingly more BECCS is built out based on the percentage of biomass removed: 25%, 50%, 75%, 90%, 99%.

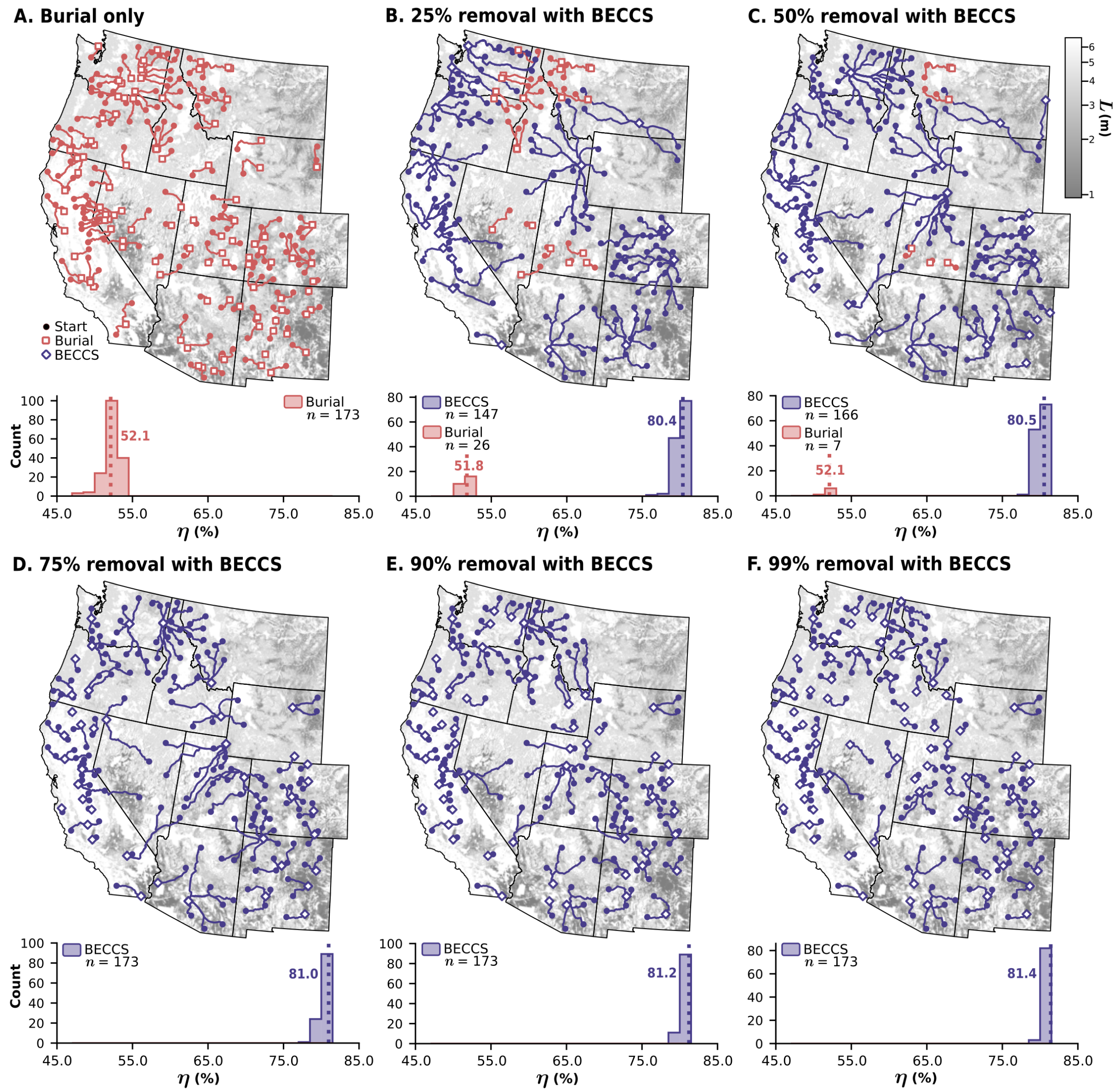

Fig. S4. Optimized modeled carbon efficiency (η) pathways for biomass burial and bioenergy with carbon capture and storage (BECCS) over six development scenarios for the low cover methane oxidation (CH_4 oxidation = 0.10) sensitivity analysis. Each panel displays (1) a map of modeled minimum soil cover thickness (***L***) overlain with the network routes between a biomass start node (solid circle) and the most carbon-efficient end node (square for burial, diamond for BECCS) for each county with thinned biomass; and (2) a histogram of $\boldsymbol{\eta}$, classified by outcome, with the labeled dotted line indicating the mean for each classification. Pathways that are optimized by burial fates are in red. Pathways optimized with BECCS fates are in blue. Each panel represents a different BECCS scenario, where **A** represents a burial-only scenario with no BECCS infrastructure, and **B-F** illustrate BECCS scenarios (*6*) where increasingly more BECCS is built out based on the percentage of biomass removed: 25%, 50%, 75%, 90%, 99%.

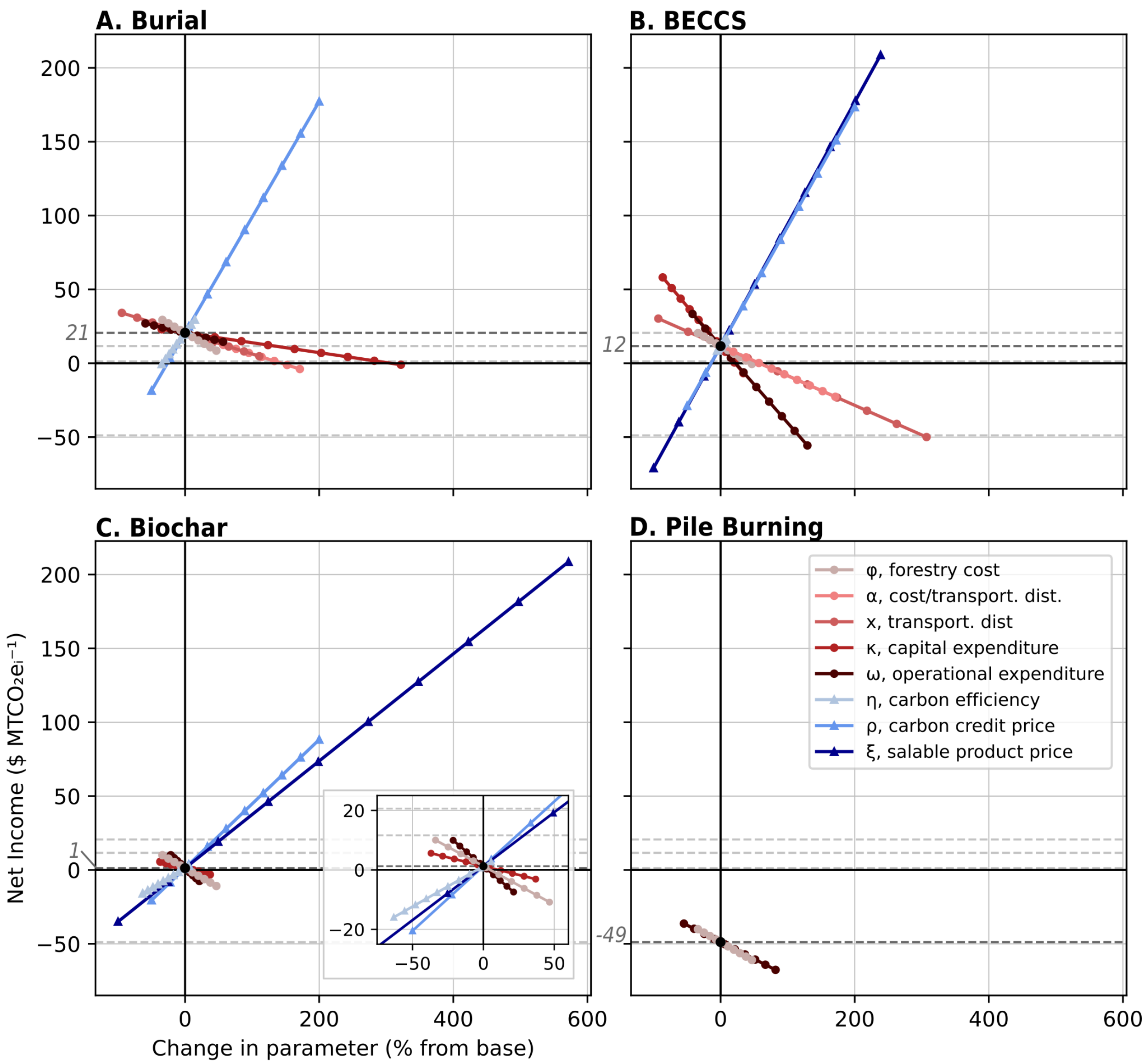

Fig. S5. Sensitivity analysis of net income per metric ton of CO_2 equivalent in initial biomass ($ $MTCO_2e_i^{-1}$) for burial (A), BECCS (B), biochar (C), and pile burning (D) outcomes, as a function of transportation cost ($\boldsymbol{\alpha}$), transportation distance ($\boldsymbol{x}$), capital expenditure ($\boldsymbol{\kappa}$), operational expenditure ($\boldsymbol{\omega}$), carbon efficiency ($\boldsymbol{\eta}$), carbon credit price ($\boldsymbol{\rho}$), and salable product price ($\boldsymbol{\xi}$). Each panel displays colored lines corresponding to the net income across the parameter space of an individual variable, with each color representing a different variable. Dark grey, dotted horizontal lines with italicized labels indicate the net income of the given pathway represented in that panel, while the light grey, dotted horizontal lines indicate the net income of the other three pathways. The absolute values for the upper and lower bounds, as well as the base case, for each parameter are listed in Table S2.

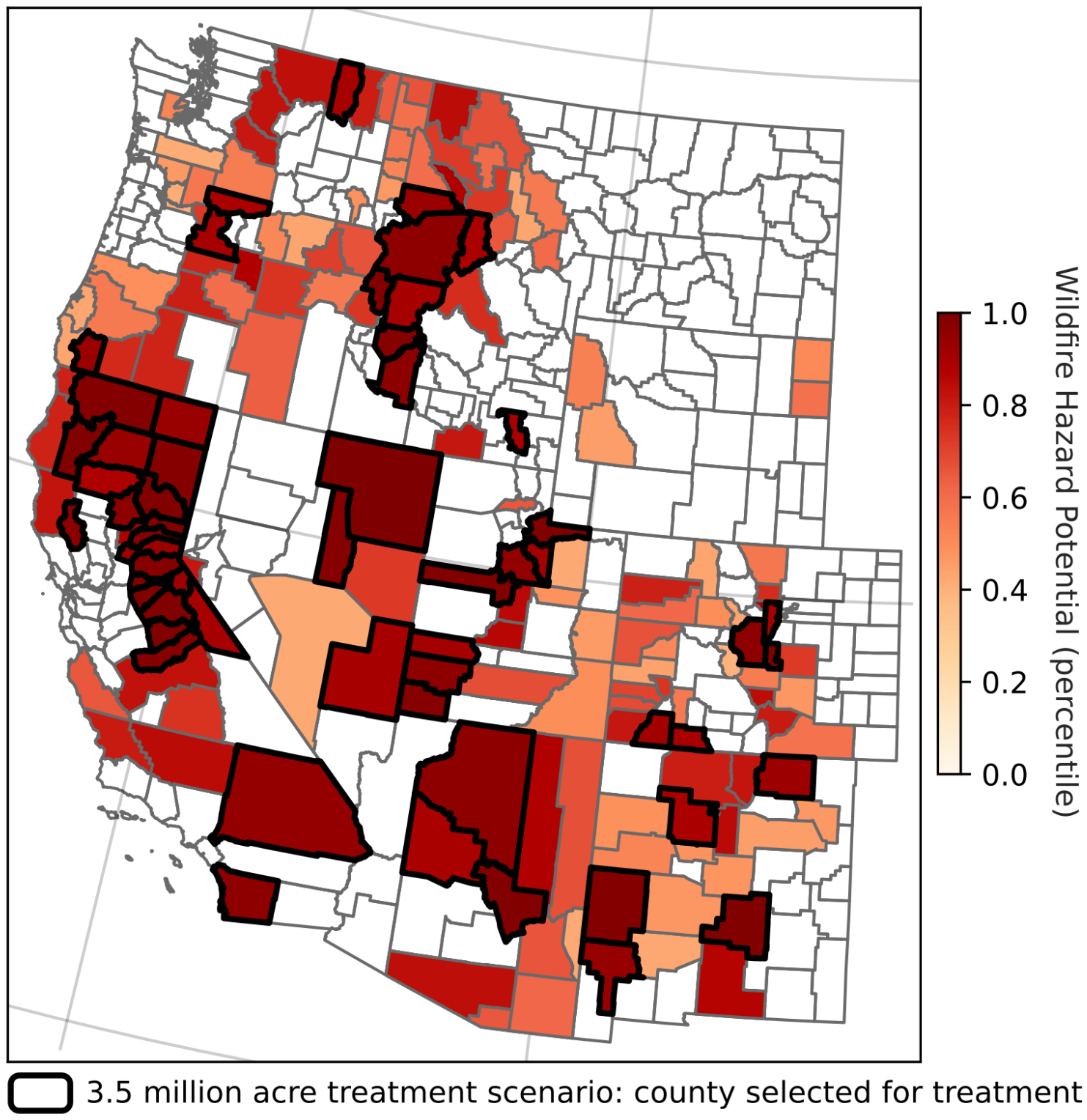

Fig. S6. Wildfire hazard potential of counties included in this study. Wildfire hazard potential describes the likelihood of high-intensity wildfire that is difficult to manage (*6*, *51*). The 57 counties of highest hazard selected under the 3.5 million acres of thinning scenario are outlined in a bold black line. Counties in white are not included in this study.

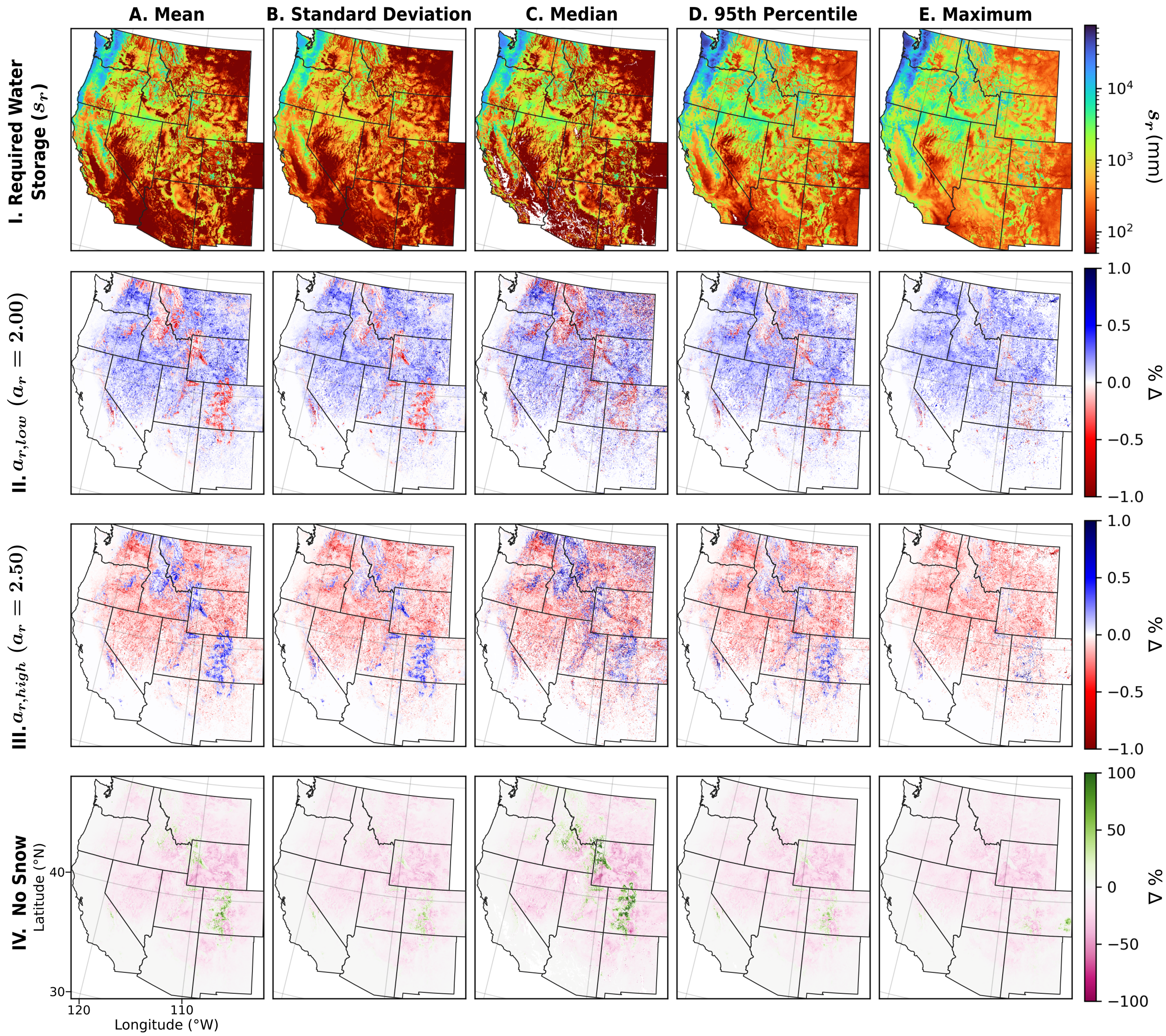

Fig. S7. Sensitivity of the required water storage (s_r, I) to snow model parameterization (II and III) and the exclusion of snow partitioning (IV). For each row, the columns correspond to the mean (**A**), standard deviation (**B**), median (**C**), 95th percentile (**D**), and maximum (**E**) values for each pixel over the 2001-2020 model period. For the 95th percentile required water storage ($s_{r,95}$), the mean percent change for the low restricted-degree day factor ($a_{r,low}$, **II**) from the base case (a_r=2.25) is 0.0459% (minimum -8.6013%, maximum 7.0120%). The mean percent change for $s_{r,95}$ for $a_{r,high}$ (**III**) is -0.0439% (minimum -8.7742%, maximum 8.6074%). For the scenario that does not partition precipitation into rainfall and snowfall and assumes that all water reaches the soil column on the day of precipitation (**IV**), the mean percent change $s_{r,95}$ is -6.534% (minimum -76.9206%, maximum 205.0774%).

Table S1. Biomass burial gate-to-grave life cycle assessment (LCA) parameters and sources. For complete calculations with formulas, see (*43*). Cells with asterisks (*) indicate variables that are selected through geospatial analysis (Materials and Methods, Section 2.2.6). Sample data are used for the example presented here. Cells with the symbol † indicate variables that we conducted sensitivity analyses on (Materials and Methods, Section 2.4.1). Headers are indicated in bold italic text. Final carbon efficiency deductions for each section are indicated in bold text.

Model Component	***Parameter***	***Unit***	***Value***	***Source***
		Material Characteristics		
Constant Value	Truck Transport Limit	metric tons	22.6796	(*15*) Table 31
Constant Value	Moisture Content (Green wood)	%	54.0000	Assumption (within range reported by (*110*))
Constant Value	Carbon content (of bone-dry wood)	%	50.0000	(*111*) For ranges, see: (*127*)
Constant Value	Conversion from short ton to metric ton	US ton:metric ton	0.9070	Unit conversion
Constant Value	Density of wood, oven-dry	metric tons per m^3	0.4450	(*128*)
Constant Value	Density of wood, 50% moisture	metric tons per m^3	0.6853	Calculated
Constant Value	C to CO_2e Conversion	mass CO_2:mass C	3.6700	Unit conversion
Constant Value	Packing Efficiency	%	50.0000	Assumption
		Process Emissions, Forestry		
Constant Value	Diesel Carbon Intensity	$MTCO_2$e per L	0.0027	(*107*) Table 2 - Mobile Combustion CO2.
Constant Value	Efficiency Multiplier	-	1.1940	(*108*)
Delimber	Delimber Hours per Green Short Ton	h per green short ton	0.0717	Mean of lower bound from field-derived estimate using Pierce PMD-3348 Stroke-Boom Delimber (*106*) and upper bound assuming efficiency multiplier from (*108*)
Delimber	Delimber Diesel Use	L per h	18.9000	Mean of lower bound from field-derived estimate using Pierce PMD-3348 Stroke-Boom Delimber (*106*) and upper bound assuming efficiency multiplier from (*108*)
**Delimber**	**Delimber emissions**	**$MTCO_2$e emitted per $MTCO_2$e in biomass**	**0.0034**	**Calculated**
Loader	Loader Hours per Green Short Ton	h per green short ton	0.0340	Mean of lower bound from field-derived estimate using Doosan DX225LL loader (*106*) and upper bound assuming efficiency multiplier from (*108*)
Loader	Loader Diesel Use	L per h	18.9000	Mean of lower bound from field-derived estimate (*106*) and upper bound assuming efficiency multiplier from (*108*)
**Loader**	**Loader emissions**	**$MTCO_2$e emitted per $MTCO_2$e in biomass**	**0.0016**	**Calculated**
Unloading / Stacking	Loader Hours per Green Short Ton	h per green short ton	0.0340	Mean of lower bound from field-derived estimate using Doosan DX225LL loader (*106*) and upper bound assuming efficiency multiplier from (*108*)
Unloading / Stacking	Loader Diesel Use	L per h	18.9000	Mean of lower bound from field-derived estimate (*106*) and upper bound assumes efficiency multiplier from (*108*)
**Unloading / Stacking**	**Loader emissions**	**$MTCO_2$e emitted per $MTCO_2$e in biomass**	**0.0016**	**Calculated**
		Transportation		
Geospatial Variable*	Distance travelled on paved roads	mi, paved roads	50.0000*	*Max is 100 miles total between paved + forestry roads*

Geospatial Variable*	Distance travelled on forestry roads	mi, forestry roads	50.0000*	*Max is 100 miles total between paved + forestry roads*
Constant Value	$MTCO_2e$ in biomass transported per truckload	$MTCO_2e$ per truckload	27.0241	Calculated
Class 8 Truck	Class 8 Truck Fuel Efficiency - Public Highway	MPG, paved public highway	6.6567	(*113*) Table A1. General Statistics for the Instrumented Tractors.
Class 8 Truck	Class 8 Truck Fuel Efficiency - Public Highway	L per truck-mile, paved public highway	0.5687	Calculated
Class 8 Truck	Class 8 Truck Fuel Efficiency - Public Highway	L per $MTCO_2e$ in biomass per mi, paved public highway	0.0210	Calculated
**Class 8 Truck**	**Paved Public Highway Transportation Emissions**	**$MTCO_2e$ emitted per $MTCO_2e$ in biomass per [# of miles], paved public highway**	**0.0001**	**Calculated**
Class 8 Truck	Class 8 Truck Fuel Efficiency - Forest Roads	MPG, forest roads	3.1550	(*114*)
Class 8 Truck	Class 8 Truck Fuel Efficiency - Forest Roads	L per truck-mile, forest roads	1.1998	Calculated
Class 8 Truck	Class 8 Truck Fuel Efficiency - Forest Roads	L per $MTCO_2e$ in biomass per mi, forest roads	0.0444	Calculated
**Class 8 Truck**	**Forest Road Transportation Emissions**	**$MTCO_2e$ emitted per $MTCO_2e$ in biomass per [# of miles], forest roads**	**0.0001**	**Calculated**
Process Emissions, Storage Chamber				
Excavator	Excavator Throughput in Cubic Yards per Hour	yd^3 per h	70.0000	Mean of field-derived estimate (lower bound) and upper bound assuming efficiency multiplier from (*108*)
Excavator	Excavator Diesel Use	L per h	49.3500	(*115*) range for 374D Large Excavator
Excavator	Excavator Fuel Efficiency	L per m^3	0.9221	Calculated
Bulldozer	Bulldozer Fuel Efficiency	L per m^3	1.5696	Mean of field-derived estimate (lower bound) and upper bound assuming efficiency multiplier from (*108*)
Geospatial Variable*	Soil cover thickness	m	2.0000*	Minimum soil cover thickness raster (L)
Excavator	Total Excavation Required (function of cover thickness)	m^3 earth moved per m^2 of storage chamber surface area	3.0000	Assumes that soil column that is excavated (in m^3 per m^2 surface area of storage chamber) is 1.5x the cover thickness to allow room for buried material
Constant Value	$MTCO^2e$ buried per square meter of storage chamber surface area	$MTCO_2e$ buried per m^2 of storage chamber surface area	1.2249	Assume 3 m^3 of buried biomass material below a 1 m^2 surface area of a storage chamber; in some cases, repacking will occur above exact level of initial plot of land
Excavator	Excavation per $MTCO^2e$ Buried	m^3 earth moved per $MTCO_2e$ buried	2.4493	Calculated
**Excavator**	**Excavator Emissions**	**$MTCO_2e$ emitted per $MTCO_2e$ in biomass**	**0.0091**	**Calculated; Assumes 1.5x excavation emissions beyond exact volume/footprint of storage chamber (conservative)**
**Bulldozer**	**Bulldozer Emissions**	**$MTCO_2e$ emitted per $MTCO_2e$ in biomass**	**0.0156**	**Calculated; Assumes 1.5x bulldozer emissions beyond exact volume/footprint of storage chamber (conservative)**

		Soil Carbon Emissions		
Geospatial Variable*	Soil Organic Matter	%	0.0500*	Location-specific from (*36*)
Soil Carbon	Soil Percent Carbon	%	0.0290	Soil Percent Carbon (%) = Soil Organic Matter (%) / 1.724 (van Bemmelen factor); (*116*)
Geospatial Variable*	Soil Bulk Density	Metric tons of soil per m^3 volume of soil	1.8000*	Location-specific from (*37*)
Soil Carbon	$MTCO_2e$ per m^3 soil	$MTCO_2e$ per m^3 soil	0.1916	Calculated
Soil Carbon	Amount of Soil $MTCO_2e$ Disturbed Per $MTCO_2e$ buried	$MTCO_2e$ of soil disturbed per $MTCO_2e$ buried	0.4693	Calculated
Soil Carbon	Oxidation Rate of Soil Carbon †	%	0.1000	Range in (*46*)
**Soil Carbon**	**Soil Carbon Emissions**	**$MTCO_2e$ emitted per $MTCO_2e$ in biomass**	**0.0469**	**Calculated**
		Emissions resulting from decay over 100 years		
Storage chamber emissions	Fraction of DOC degradable in anoxic conditions, DOC_f †	proportion	0.0880	(*26*) Table 3.0 (page 3.12); lower bound (0.03) from (*44*) and citations within; upper bound (0.183) hardwood average from (*13*)6/30/26 2:28:00 PM
Storage chamber emissions	Biomass Decay Emissions, CO_2	$MTCO_2e$ per $MTCO_2e$ in biomass	0.0440	Assumes full decay of DOC_f into equimolar parts CO_2 and CH_4
Storage chamber emissions	Biomass Decay Emissions, CH_4 (if no oxidation in soil layer)	$MTCO_2e$ per $MTCO_2e$ in biomass	0.4476	Assumes full decay of DOC_f into equimolar parts CO_2 and CH_4assumes 28 as GWP_{100} of CH_4
Storage chamber emissions	Oxidation of CH_4 in Soil Layer †	proportion	0.7500	Central estimate (0.75) derived from: (*45*) ET cover oxidation rate of 62% but higher rates of CH_4 generation; and (*33*) oxidation rate can be up to 100% and efficiency is far higher when the flux of CH_4 into the cover is low, representative of ET covers
**Storage Chamber emissions**	**Biomass Decay Emissions, total**	**$MTCO_2e$ emitted per $MTCO_2e$ in biomass, 100 years**	**0.1889**	**Calculated; Assumes that CH_4 oxidized to CO_2 in soil cover is emitted as CO_2; full decay of all DOC_f**
		Final Result		
**Total**	**Carbon Efficiency of Burial**	**$MTCO_2e$ emitted per $MTCO_2e$ in biomass, 100 years, as %**	**73.3%**	**Calculated; 1 minus all deductions indicated above**

Table S2. Technoeconomic analysis (TEA) parameters, units, and sources. All $ values are in 2026 United States dollars.

Parameter	Pathway	Variable	Range	Value	Unit	Sources
Forest processes	All	φ	Low	17.17	$ $MTCO_2e_i^{-1}$	(*119*, *120*)
			Base	25.93	$ $MTCO_2e_i^{-1}$	(*119*, *120*)
			Upper	38.03	$ $MTCO_2e_i^{-1}$	(*119*, *120*)
Transportation cost	Burial, BECCS	α	Low, Base	0.1023	$ $MTCO_2e_i^{-1}$ km^{-1}	(*48*)
			Upper	0.2774	$ $MTCO_2e_i^{-1}$ km^{-1}	Theoretical margin
One-way transportation distance	Burial	x_b	Low	8	km	This study, minimum
			Base	140	km	This study, mean
			Upper	295	km	This study, maximum
	BECCS	x_B	Low	14	km	This study, minimum
			Base	196	km	This study, mean
			Upper	798	km	This study, maximum
Carbon efficiency	Burial	η_b	Low	0.511	$MTCO_2e_s$ $MTCO_2e_i^{-1}$	This study, low CH_4 oxidation scenario mean minus 0.01 for transportation
			Base	0.783	$MTCO_2e_s$ $MTCO_2e_i^{-1}$	This study, mean base case
			Upper	0.897	$MTCO_2e_s$ $MTCO_2e_i^{-1}$	This study, low DOC_f scenario mean minus 0.01 for transportation
	BECCS	η_B	Low	0.767	$MTCO_2e_s$ $MTCO_2e_i^{-1}$	This study, lowest individual BECCS facility
			Base	0.809	$MTCO_2e_s$ $MTCO_2e_i^{-1}$	This study, mean
			Upper	0.878	$MTCO_2e_s$ $MTCO_2e_i^{-1}$	(*14*)
	Biochar	η_c	Low	0.160	$MTCO_2e_s$ $MTCO_2e_i^{-1}$	(*39*)
			Base	0.435	$MTCO_2e_s$ $MTCO_2e_i^{-1}$	This study
			Upper	0.459	$MTCO_2e_s$ $MTCO_2e_i^{-1}$	(*124*)
Capital expenditure	Burial	κ_b	Low	5.585	$ $MTCO_2e_s^{-1}$	(*16*, *19*)
			Base	8.58	$ $MTCO_2e_s^{-1}$	(*16*, *19*)
			Upper	36.18	$ $MTCO_2e_s^{-1}$	Theoretical margin
	BECCS	κ_B	Low	9.15	$ $MTCO_2e_s^{-1}$	(*6*)
			Base	66.64	$ $MTCO_2e_s^{-1}$	(*6*)
			Upper	89.27	$ $MTCO_2e_s^{-1}$	(*6*)
	Biochar	κ_c	Low	17.09	$ $MTCO_2e_s^{-1}$	Theoretical margin
			Base	27.09	$ $MTCO_2e_s^{-1}$	(*123*)
			Upper	37.09	$ $MTCO_2e_s^{-1}$	Theoretical margin
Operational expenditure	Burial	ω_b	Low	5.59	$ $MTCO_2e_s^{-1}$	(*19*)
			Base	13.68	$ $MTCO_2e_s^{-1}$	(*16*, *19*)
			Upper	21.44	$ $MTCO_2e_s^{-1}$	Theoretical margin
	BECCS	ω_B	Low	37.34	$ $MTCO_2e_s^{-1}$	(*6*)
			Base	64.24	$ $MTCO_2e_s^{-1}$	(*6*)
			Upper	147.40	$ $MTCO_2e_s^{-1}$	(*6*)
	Biochar	ω_c	Low	73.78	$ $MTCO_2e_s^{-1}$	Theoretical margin
			Base	93.78	$ $MTCO_2e_s^{-1}$	(*123*)
			Upper	113.78	$ $MTCO_2e_s^{-1}$	Theoretical margin
	Pile burning	ω_p	Low	10.38	$ $MTCO_2e_i^{-1}$	(*121*)
			Base	22.91	$ $MTCO_2e_i^{-1}$	(*10*)
			Upper	41.69	$ $MTCO_2e_i^{-1}$	(*10*)
Carbon credit price	Burial, BECCS, biochar	ρ	Low	50.00	$ $MTCO_2e_s^{-1}$	(*6*)
			Base	100.00	$ $MTCO_2e_s^{-1}$	(*6*)
			Upper	300.00	$ $MTCO_2e_s^{-1}$	(*6*)
Salable product price	BECCS	ξ_B	Low	0.00	$ $MTCO_2e_s^{-1}$	No product market
			Base	102.09	$ $MTCO_2e_s^{-1}$	(*6*)
			Upper	345.69	$ $MTCO_2e_s^{-1}$	(*6*, *47*, *125*)
	Biochar	ξ_c	Low	0.00	$ $MTCO_2e_s^{-1}$	No product market
			Base	83.36	$ $MTCO_2e_s^{-1}$	(*123*)
			Upper	560.00	$ $MTCO_2e_s^{-1}$	(*124*)

REFERENCES

1. J. T. Abatzoglou, C. A. Kolden, A. C. Cullen, M. Sadegh, E. L. Williams, M. Turco, M. W. Jones, Climate change has increased the odds of extreme regional forest fire years globally. *Nat. Commun.* **16**, 6390 (2025).

2. X.-L. Yue, Q.-X. Gao, Contributions of natural systems and human activity to greenhouse gas emissions. *Adv. Clim. Chang. Res.* **9**, 243–252 (2018).

3. P. F. Hessburg, S. J. Prichard, R. K. Hagmann, N. A. Povak, F. K. Lake, Wildfire and climate change adaptation of western North American forests: A case for intentional management. *Ecol. Appl.* **31**, e02432 (2021).

4. P. Z. Fulé, J. E. Crouse, J. P. Roccaforte, E. L. Kalies, Do thinning and/or burning treatments in western USA ponderosa or Jeffrey pine-dominated forests help restore natural fire behavior?. *For. Ecol. Manage.* **269**, 68–81 (2012).

5. US Department of Agriculture (USDA) Forest Service, "Confronting the wildfire crisis" (FS-1187a, USDA Forest Service, 2022); www.fs.usda.gov/sites/default/files/fs_media/fs_document/Confronting-the-Wildfire-Crisis.pdf.

6. J. Pett-Ridge, S. Kuebbing, A. Mayer, S. Hovorka, H. Pilorgé, S. Baker, S. Pang, C. Scown, K. Mayfield, A. Wong, R. Aines, H. Ammar, A. Aui, M. Ashton, B. Basso, M. Bradford, A. Bump, I. Busch, E. Calzado, J. Chirigotis, N. Clauser, S. Crotty, N. Dahl, T. Dai, M. Ducey, J. Dumortier, N. Ellebracht, R. Egui, A. Fowler, K. Georgiou, D. Giannopoulos, H. Goldstein, T. Harris, D. Hayes, C. Hellwinckel, A. Ho, M. Hong, E. Hunter-Sellars, W. Kirkendall, M. Langholtz, M. Layer, I. Lee, R. Lewis, W. Li, W. Liu, J. Lozano, A. Lunstrum, W. McNeil, P. Nico, A. O'Rourke, K. Paustian, G. Peridas, M. Pisciotta, L. Price, P. Psarras, G. Robertson, W. Sagues, D. Sanchez, B. Schmidt, E. Slessarev, N. Sokol, A. Stanley, A. Swan, C. Toureene, M. Wright, Y. Yao, B. Zhang, Y. Zhang, "Roads to Removal: Options for carbon dioxide removal in the United States" (LLNL--TR-852901, 2301853, 1080440, 2023); https://doi.org/10.2172/2301853.

7. K. T. Davis, J. Peeler, J. Fargione, R. D. Haugo, K. L. Metlen, M. D. Robles, T. Woolley, Tamm review: A meta-analysis of thinning, prescribed fire, and wildfire effects on subsequent wildfire severity in conifer dominated forests of the Western US. *For. Ecol. Manage.* **561**, 121885 (2024).

8. B. Springsteen, T. Christofk, S. Eubanks, T. Mason, C. Clavin, B. Storey, Emission reductions from woody biomass waste for energy as an alternative to open burning. *J. Air Waste Manag. Assoc.* **61**, 63–68 (2011).

9. K. R. Fingerman, J. Qiriazi, C. L. Barrientos, M. Blasdel, J. M. Comnick, A. R. Harris, C. Geronimo, C.-W. Hsu, J. M. Kane, E. Oneil, S. Rios-Romero, L. W. Rogers, M. Severy, M. C. Wright, Climate and air pollution impacts of generating biopower from forest management residues in California. *Environ. Res. Lett.* **18**, 034038 (2023).

10. J. Barker, J. Voorhis, S. M. Crotty, Assessing costs and constraints of forest residue disposal by pile burning. *Front. For. Glob. Change* **7**, 1496190 (2025).

11. P. Khatri, R. Bergman, P. Nepal, D. Nicholls, A. Gray, Evaluating life cycle greenhouse gas emissions from alternative forest residue utilization: Energy and product pathways. *Biomass Bioenergy* **201**, 108059 (2025).

12. D. Sandalow, R. Aines, J. Friedmann, C. McCormick, D. Sanchez, “Biomass carbon removal and storage (BiRCS) roadmap” (LLNL-TR-815200, Lawrence Livermore National Laboratory, 2021); https://doi.org/10.2172/1763937.

13. S. M. Crotty, P. W. Reiners, L. K. Clayton, E. Young, A. Jones, M. A. Cregger, A. K. Starace, A. E. Harman-Ware, Nonenergy biomass carbon removal and storage (BiCRS): Assessing durability of nongaseous carbon products across terrestrial storage fates. *Chem. Rev.* **126**, 4375–4404 (2026).

14. D. L. Sanchez, P. Psarras, H. Murnen, B. Rogers, Carbon removal efficiency and energy requirement of engineered carbon removal technologies. *RSC Sustain.* **3**, 1424–1433 (2025).

15. S. E. Baker, J. K. Stolaroff, G. Peridas, S. H. Pang, H. M. Goldstein, F. R. Lucci, W. Li, E. W. Slessarev, J. Pett-Ridge, F. J. Ryerson, J. L. Wagoner, W. Kirkendall, R. D. Aines, D. L. Sanchez, B. Cabiyo, J. Baker, S. McCoy, S. Uden, R. Runnebaum, J. Wilcox, P. C. Psarras, H. Pilorge, N. McQueen, D. Maynard, C. McCormick, "Getting to neutral: Options for negative carbon emissions in California" (LLNL-TR--796100, Lawrence Livermore National Laboratory; University of California, Berkeley; Negative Carbon Consulting; University of Calgary; University of Queensland; University of California, Davis; Worcester Polytechnic Institute; Georgetown University; Valence Strategic, 2019); https://doi.org/10.2172/1597217.

16. E. Yablonovitch, H. W. Deckman, Scalable, economical, and stable sequestration of agricultural fixed carbon. *Proc. Natl. Acad. Sci. U.S.A.* **120**, e2217695120 (2023).

17. N. Zeng, X. Zhao, G. Poisson, B. Clifford, Y. Liu, H. Liu, T. Meng, L. Picard, E. Zeng-Mariotti, B. Zaitchik, L. Hu, 3775-year-old wood burial supports "wood vaulting" as a durable carbon removal method. *Science* **385**, 1454–1459 (2024).

18. Y. Luo, N. Wei, X. Lu, Y. Zhou, F. Tao, Q. Quan, C. Liao, L. Jiang, J. Xia, Y. Huang, S. Niu, X. Xu, Y. Sun, N. Zeng, C. Koven, L. Peng, S. Davis, P. Smith, F. You, Y. Jiang, L. Cheng, B. Houlton, Large CO_2 removal potential of woody debris preservation in managed forests. *Nat. Geosci.* **18**, 675–681 (2025).

19. N. Zeng, H. Hausmann, Wood Vault: Remove atmospheric CO_2 with trees, store wood for carbon sequestration for now and as biomass, bioenergy and carbon reserve for the future. *Carbon Balance Manag.* **17**, 2 (2022).

20. M. Bittelli, G. S. Campbell, F. Tomei, *Soil Physics with Python: Transport in the Soil–Plant–Atmosphere System* (Oxford Univ. Press, 2015).

21. Z. Liu, J. S. Kimball, A. Ballantyne, J. D. Watts, S. M. Natali, B. M. Rogers, Y. Yi, A. E. Klene, M. Moghaddam, J. Du, D. Zona, Widespread deepening of the active layer in northern permafrost regions from 2003 to 2020. *Environ. Res. Lett.* **19**, 014020 (2023).

22. L. Feng, J. Hu, J. Jiang, Uncertainty in soil gas permeability measurements. *Front. Environ. Sci.* **13**, (2025).

23. R. Cossu, R. Stegmann, *Solid Waste Landfilling: Concepts, Processes, Technology* (Elsevier, 2018).

24. C. M. Bethke, R. A. Sanford, M. F. Kirk, Q. Jin, T. M. Flynn, The thermodynamic ladder in geomicrobiology. *Am. J. Sci.* **311**, 183–210 (2011).

25. IPCC, *Climate Change 2023: Synthesis Report: Contribution of Working Groups I, II and III to the Sixth Assessment Report of the Intergovernmental Panel on Climate Change* (IPCC, 2023).

26. S. Towprayoon, T. Ishigaki, C. Chiemchaisri, A. O. Abdel-Aziz, *2019 Refinement to the 2006 IPCC Guidelines for National Greenhouse Gas Inventories, Chapter 3: Solid Waste Disposal* (IPCC, 2019).

27. H. H. Hilger, M. A. Barlaz, "Anaerobic decomposition of refuse in landfills and methane oxidation in landfill covers," in *Manual of Environmental Microbiology* (John Wiley & Sons Ltd., 2007), pp. 818–842.

28. X. Wang, M. A. Barlaz, Decomposition and carbon storage of hardwood and softwood branches in laboratory-scale landfills. *Sci. Total Environ.* **557–558**, 355–362 (2016).

29. A. Stevenson, J. A. Cray, J. P. Williams, R. Santos, R. Sahay, N. Neuenkirchen, C. D. McClure, I. R. Grant, J. D. Houghton, J. P. Quinn, D. J. Timson, S. V. Patil, R. S. Singhal, J. Antón, J. Dijksterhuis, A. D. Hocking, B. Lievens, D. E. N. Rangel, M. A. Voytek, N. Gunde-Cimerman, A. Oren, K. N. Timmis, T. J. McGenity, J. E. Hallsworth, Is there a common water-activity limit for the three domains of life? *ISME J.* **9**, 1333–1351 (2015).

30. H. E. Beck, T. R. McVicar, N. Vergopolan, A. Berg, N. J. Lutsko, A. Dufour, Z. Zeng, X. Jiang, A. I. J. M. van Dijk, D. G. Miralles, High-resolution (1 km) Köppen-Geiger maps for 1901–2099 based on constrained CMIP6 projections. *Sci. Data* **10**, 724 (2023).

31. W. H. Albright, C. H. Benson, W. J. Waugh, *Water Balance Covers for Waste Containment: Principles and Practice* (American Society of Civil Engineers, 2010).

32. P. Kjeldsen, M. A. Barlaz, A. P. Rooker, A. Baun, A. Ledin, T. H. Christensen, Present and long-term composition of MSW landfill leachate: A review. *Crit. Rev. Environ. Sci. Technol.* **32**, 297–336 (2002).

33. J. Chanton, T. Abichou, C. Langford, K. Spokas, G. Hater, R. Green, D. Goldsmith, M. A. Barlaz, Observations on the methane oxidation capacity of landfill soils. *Waste Manag.* **31**, 914–925 (2011).

34. Soil Survey Staff, Gridded National Soil Survey Geographic (gNATSGO) Database for the Conterminous United States, version FY2024, US Department of Agriculture, Natural Resources Conservation Service (2023); https://nrcs.app.box.com/v/soils.

35. O. Boiko, S. Kagone, G. Senay, Soil properties dataset in the United States, US Geological Survey (2021); https://doi.org/10.5066/P9TI3IS8.

36. G. Boeing, Modeling and analyzing urban networks and amenities with OSMnx. *Geog. Anal.* **57**, 567–577 (2025).

37. S. Chiquier, P. Patrizio, M. Bui, N. Sunny, N. M. Dowell, A comparative analysis of the efficiency, timing, and permanence of CO_2 removal pathways. *Energy Environ. Sci.* **15**, 4389–4403 (2022).

38. F. J. Cook, J. H. Knight, F. M. Kelliher, Modelling oxygen transport in soil with plant root and microbial oxygen consumption: Depth of oxygen penetration. *Soil Res.* **51**, 539–553 (2013).

39. S. Low, S. Schäfer, Is bio-energy carbon capture and storage (BECCS) feasible? The contested authority of integrated assessment modeling. *Energy Res. Soc. Sci.* **60**, 101326 (2020).

40. A. Almena, V. G. Pashakolaie, M. Martin, Unlocking BECCS viability through monetizing societal benefits by techno-socio-economic assessment. *Sustainable Prod. Consumption* **59**, 255–273 (2025).

41. L. K. Clayton, leah-clayton/biomass-carbon-storage: v1.1, Zenodo (2026); https://doi.org/10.5281/zenodo.17645349.

42. D. Johnson, J. Voorhis, S. Porder, Life cycle emissions associated with vault storage of wood cleared for fire management in the Western United States. *Carbon Balance Manag.* **20**, 26 (2025).

43. US Department of Energy Office of Critical Minerals and Energy Innovation, “Guidelines to determine well-to-gate greenhouse gas (GHG) emissions of hydrogen production pathways using 45VH2-GREET (2025).

44. J. K. Stolaroff, S. H. Pang, W. Li, W. G. Kirkendall, H. M. Goldstein, R. D. Aines, S. E. Baker, Transport cost for carbon removal projects with biomass and CO_2 storage. *Front. Energy Res.* **9**, 639943 (2021).

45. E. E. Hjerpe, P. A. Champ, S. G. Evans, J. J. Sánchez, K. W. Jones, J. Loomis, T. P. Holmes, G. Aldrich, M. Buckley, M. M. Colavito, A. Sánchez Meador, Economic efficiency of fuel treatments in western US forests: A methodological framework. *Int. J. Wildland Fire* **35**, WF25060 (2026).

46. US Wildland Fire Service, “Budget justifications and performance information FY 2026: US Wildland Fire Service” (Budget Justifications and Performance Information, US Department of the Interior, 2025); www.doi.gov/sites/default/files/documents/2025-07/uswfs-2026-greenbook508.pdf.

47. G. K. Dillon, J. H. Scott, M. R. Jaffe, J. H. Olszewski, K. C. Volger, M. A. Finney, K. C. Short, K. L. Riley, I. C. Grenfell, W. M. Jolly, S. Brittain, Spatial datasets of probabilistic wildfire risk components for the United States (270m), version 3rd Edition, Forest Service Research Data Archive (2023); https://doi.org/10.2737/RDS-2016-0034-3.

48. S. J. Prichard, R. D. Ottmar, G. K. Anderson, “Consume 3.0 user’s guide” (USDA Forest Service, Pacific Northwest Research Station, Pacific Wildland Fire Sciences Laboratory, Fire and Environmental Research Applications Team, 2006); www.frames.gov/catalog/1260.

49. G. A. Woolsey, W. T. Tinkham, M. A. Battaglia, C. M. Hoffman, Constraints on mechanical fuel reduction treatments in United States Forest Service Wildfire Crisis Strategy priority landscapes. *J. For.* **122**, 335–351 (2024).

50. H. Chang, H.-S. Han, N. Anderson, Y.-S. Kim, S.-K. Han, The cost of forest thinning operations in the western United States: A systematic literature review and new thinning cost model. *J. For.* **121**, 193–206 (2023).

51. D. Pierson, N. Anderson, J. Brewen, N. Clark, M. C. Hardy, D. McCollum, F. H. McCormick, J. Morisette, T. Nicosia, D. Page-Dumroese, C. Rodriguez-Franco, J. Tirocke, Beyond the basics: A perspective on barriers and opportunities for scaling up biochar production from forest slash. *Biochar* **6**, 1 (2024).

52. S. Fuss, W. F. Lamb, M. W. Callaghan, J. Hilaire, F. Creutzig, T. Amann, T. Beringer, W. de Oliveira Garcia, J. Hartmann, T. Khanna, G. Luderer, G. F. Nemet, J. Rogelj, P. Smith, J. L. V. Vicente, J. Wilcox, M. del Mar Zamora, J. C. Dominguez, J. C. Minx, Negative emissions—Part 2: Costs, potentials and side effects. *Environ. Res. Lett.* **13**, 063002 (2018).

53. C. Kooistra, C. Schultz, J. Abrams, H. Huber-Stearns, Institutionalizing the United States Forest Service's Shared Stewardship Strategy in the Western United States. *J. For.* **120**, 588–603 (2022).

54. J. W. Nyhan, A seven-year water balance sudy of an evapotranspiration landfill cover varying in slope for semiarid regions. *Vadose Zone J.* **4**, 466–480 (2005).

55. Y. Fan, G. Miguez-Macho, E. G. Jobbágy, R. B. Jackson, C. Otero-Casal, Hydrologic regulation of plant rooting depth. *Proc. Natl. Acad. Sci. U.S.A.* **114**, 10572–10577 (2017).

56. J. M. Simunek, M. T. Van Genuchten, M. Sejna, The HYDRUS-1D software package for simulating the one-dimensional movement of water, heat, and multiple solutes in variably-saturated media. *UC-Riverside Res. Rep.* **3**, 240 (2005).

57. M. J. Fayer, “UNSAT-H version 3.0: Unsaturated soil water and heat flow model theory, user manual, and examples” (820201000, Pacific Northwest National Laboratory, 2000); https://doi.org/10.2172/781853.

58. R. G. Striegl, T. A. McConnaughey, D. C. Thorstenson, E. P. Weeks, J. C. Woodward, Consumption of atmospheric methane by desert soils. *Nature* **357**, 145–147 (1992).

59. K. J. Sparrow, J. P. Chanton, R. B. Green, C. Scheutz, G. R. Hater, L. C. Wilson, T. Abichou, Stable isotopic determination of methane oxidation: When smaller scales are better. *Waste Manag.* **97**, 82–87 (2019).

60. J. Gebert, M. Huber-Humer, A. R. Cabral, Design of microbial methane oxidation systems for landfills. *Front. Environ. Sci.* **10**, (2022).

61. J. Filer, H. H. Ding, S. Chang, Biochemical methane potential (BMP) assay method for anaerobic digestion research. *Water* **11**, 921 (2019).

62. A. P. Williams, E. R. Cook, J. E. Smerdon, B. I. Cook, J. T. Abatzoglou, K. Bolles, S. H. Baek, A. M. Badger, B. Livneh, Large contribution from anthropogenic warming to an emerging North American megadrought. *Science* **368**, 314–318 (2020).

63. I. R. Simpson, K. A. McKinnon, D. Kennedy, D. M. Lawrence, F. Lehner, R. Seager, Observed humidity trends in dry regions contradict climate models. *Proc. Natl. Acad. Sci. U.S.A.* **121**, e2302480120 (2024).

64. J. Ye, Z. Wang, An origin of North American monsoon retreat biases in climate models. *J. Climate* **36**, 4665–4676 (2023).

65. D. L. Swain, A. F. Prein, J. T. Abatzoglou, C. M. Albano, M. Brunner, N. S. Diffenbaugh, D. Singh, C. B. Skinner, D. Touma, Hydroclimate volatility on a warming Earth. *Nat. Rev. Earth Environ.* **6**, 35–50 (2025).

66. D. L. Swain, J. T. Abatzoglou, C. Kolden, K. Shive, D. A. Kalashnikov, D. Singh, E. Smith, Climate change is narrowing and shifting prescribed fire windows in western United States. *Commun. Earth Environ.* **4**, 340 (2023).

67. R. H. Nolan, L. Collins, A. Leigh, M. K. J. Ooi, T. J. Curran, T. A. Fairman, V. R. de Dios, R. Bradstock, Limits to post-fire vegetation recovery under climate change. *Plant Cell Environ.* **44**, 3471–3489 (2021).

68. T. DeVries, F. Primeau, C. Deutsch, The sequestration efficiency of the biological pump. *Geophys. Res. Lett.* **39**, (2012).

69. M. Lessmann, G. H. Ros, M. D. Young, W. de Vries, Global variation in soil carbon sequestration potential through improved cropland management. *Glob. Chang. Bio.* **28**, 1162–1177 (2022).

70. S. L. Brantley, Understanding the lab-field discrepancy in mineral dissolution from flasks to enhanced rock weathering. *Rev. Geophys.* **63**, e2025RG000881 (2025).

71. N. G. McDowell, C. D. Allen, K. Anderson-Teixeira, B. H. Aukema, B. Bond-Lamberty, L. Chini, J. S. Clark, M. Dietze, C. Grossiord, A. Hanbury-Brown, G. C. Hurtt, R. B. Jackson, D. J. Johnson, L. Kueppers, J. W. Lichstein, K. Ogle, B. Poulter, T. A. M. Pugh, R. Seidl, M. G. Turner, M. Uriarte, A. P. Walker, C. Xu, Pervasive shifts in forest dynamics in a changing world. *Science* **368**, eaaz9463, (2020).

72. D. E. Calkin, M. P. Thompson, M. A. Finney, Negative consequences of positive feedbacks in US wildfire management. *For. Ecosyst.* **2**, 9 (2015).

73. B. Cabiyo, F. Chay, C. Field, K. Fingerman, Z. Hausfather, K. S. Hemes, C. M. Zarakas, Consistent temporal accounting supports credible CDR use. CDRXIV [Preprint] (2026). https://doi.org/10.70212/cdrxiv.2026302v1.

74. F. Strabo, C. Bryan, M. N. Reimer, Wildfire damages and the cost-effective role of forest fuel treatments. *Science* **392**, 629–635 (2026).

75. J. G. Bockheim, A. E. Hartemink, Distribution and classification of soils with clay-enriched horizons in the USA. *Geoderma* **209-210**, 153–160 (2013).

76. Q. Tang, L. Lu, Y. Xiao, D. Wang, Z. Li, Microbial interaction networks as climate thermometers: Redefining temperature sensitivity of aerobic methanotrophy in freshwater ecosystems. *npj Biodivers.* **5**, 8 (2026).

77. H. Xie, X. Zuo, H. Yan, Y. Peng, X. Gu, Y. Chen, Y. Chen, A steady-state analytical model for coupled methane transport and oxidation in vegetated landfill cover soil. *Comput. Geotech.* **152**, 105006 (2022).

78. M. M. Thornton, R. Shrestha, Y. Wei, P. E. Thornton, S.-C. Kao, Daymet: Daily surface weather data on a 1-km grid for North America, version 4 R1, ORNL DAAC (2022); https://doi.org/10.3334/ORNLDAAC/2129.

79. G. B. Senay, S. Kagone, Daily SSEBop evapotranspiration: US Geological Survey Data Release, US Geological Survey (2019); https://doi.org/10.5066/P9L2YMV.

80. D. A. Cameron, The extent of soil desiccation near trees in a semi-arid environment. *Geotech. Geol. Eng.* **19**, 357–370 (2001).

81. L. C. Torres, T. Keller, R. P. de Lima, C. A. Tormena, H. V. de Lima, N. F. B. Giarola, Impacts of soil type and crop species on permanent wilting of plants. *Geoderma* **384**, 114798 (2021).

82. M. V. Khire, C. H. Benson, P. J. Bosscher, Field data from a capillary barrier and model predictions with UNSAT-H. *J. Geotech. Geoenviron. Eng.* **125**, 518 (1999).

83. P. E. Thornton, R. Shrestha, M. Thornton, S.-C. Kao, Y. Wei, B. E. Wilson, Gridded daily weather data for North America with comprehensive uncertainty quantification. *Sci. Data* **8**, 190 (2021).

84. T. Chegini, H.-Y. Li, L. R. Leung, HyRiver: Hydroclimate data retriever. *J. Open Source Softw.* **6**, 3175 (2021).

85. W. P. Kustas, A. Rango, R. Uijlenhoet, A simple energy budget algorithm for the snowmelt runoff model. *Water Resour. Res.* **30**, 1515–1527 (1994).

86. J. Martinec, "Hour-to-hour snowmelt rates and lysimeter outflow during an entire ablation period," in *Snow Cover and Glacier Variations*. *Proceedings of the Baltimore Symposium*, A. Rango, Ed. (IAHS Publications, 1989), vol. 183, pp. 19–28.

87. P. G. Curley, E. E. Adams, L. R. McKittrick, "The influence of solar radiation attenuation in snow on near surface energy balance in complex topography," in *Proceedings of the International Snow Science Workshop 2014* (International Snow Science Workshop, 2014), pp. 567–573.

88. M. V. Khire, C. H. Benson, P. J. Bosscher, Water balance modeling of earthen final covers. *J. Geotech. Geoenviron. Eng.* **123**, 744–754 (1997).

89. G. B. Senay, S. Bohms, R. K. Singh, P. H. Gowda, N. M. Velpuri, H. Alemu, J. P. Verdin, Operational evapotranspiration mapping using remote sensing and weather datasets: A new parameterization for the SSEB approach. *J. Am. Water Resour. Assoc.* **49**, 577–591 (2013).

90. G. B. Senay, Satellite psychrometric formulation of the operational simplified surface energy balance (SSEBop) model for quantifying and mapping evapotranspiration. *Appl. Eng. Agric.* **34**, 555–566 (2018).

91. R. J. Zomer, J. Xu, A. Trabucco, Version 3 of the global aridity index and potential evapotranspiration database. *Sci. Data* **9**, 409 (2022).

92. M. P. Hiestand, H. J. Tollerud, C. Funk, G. B. Senay, K. C. Fickas, M. O. Friedrichs, SSEBop evapotranspiration estimates using synthetically derived landsat data from the continuous change detection and classification algorithm. *Remote Sens.* **16**, 1297 (2024).

93. S. T. Jackson, J. T. Overpeck, Responses of plant populations and communities to environmental changes of the late quaternary. *Paleobiology* **26**, 194–220 (2000).

94. T. Hengl, J. M. de Jesus, G. B. M. Heuvelink, M. R. Gonzalez, M. Kilibarda, A. Blagotić, W. Shangguan, M. N. Wright, X. Geng, B. Bauer-Marschallinger, M. A. Guevara, R. Vargas, R. A. MacMillan, N. H. Batjes, J. G. B. Leenaars, E. Ribeiro, I. Wheeler, S. Mantel, B. Kempen, SoilGrids250m: Global gridded soil information based on machine learning. *PLOS ONE* **12**, e0169748 (2017).

95. W. O. Zell, W. E. Sanford, Calibrated simulation of the long-term average surficial groundwater system and derived spatial distributions of its characteristics for the contiguous united states. *Water Resour. Res.* **56**, e2019WR026724 (2020).

96. W. O. Zell, W. E. Sanford, MODFLOW 6 models used to simulate the long-term average surficial groundwater system for the contiguous United States. *U.S. Geol. Surv. Data Release*, 10.5066/P91LFFN1 (2020).

97. USGS, Annual NLCD Collection 1 Science Products (2024); https://doi.org/10.5066/P94UXNTS.

98. USGS, Protected Areas Database of the United States (PAD-US) 4, *U.S. Geol. Surv. Data Release* 10.5066/P96WBCHS (2025).

99. M. B. Russell, C. W. Woodall, S. Fraver, A. W. D'Amato, G. M. Domke, K. E. Skog, Residence times and decay rates of downed woody debris biomass/carbon in eastern US forests. *Ecosystems* **17**, 765–777 (2014).

100. D. V. Sandberg, "Research leads to less smoke from prescribed fires," in *Proceedings of the 1983 Annual Meeting of the Northwest Forest Fire Council* (Northwest Fire Council, 1983), pp. 1–15.

101. D. E. Ward, C. C. Hardy, D. V. Sandberg, T. E. Reinhard, "Mitigation of prescribed fire atmospheric pollution through increased utilization of hardwoods, piled residues, and long-needled conifers: Final report" [IAG DE-AI179-85BPI8509 (PNW-85-423), United States Forest Service, Pacific Northwest Research Station, 1989]; https://nfsl.contentdm.oclc.org/digital/collection/p17053coll2/id/1576/.

102. S. J. Prichard, M. C. Kennedy, Fuel treatment effects on tree mortality following wildfire in dry mixed conifer forests, Washington State, USA. *Int. J. Wildland Fire* **21**, 1004–1013 (2012).

103. C. C. Hardy, “Guidelines for estimating volume, biomass, and smoke production for piled slash” (Gen. Tech. Rep. PNW-GTR-364, US Department of Agriculture, Forest Service, Pacific Northwest Research Station, 1996), pp. 1–17.

104. K. Systems, “Carbon dioxide removal prepurchase application” (Frontier Climate, 2022); https://github.com/frontierclimate/carbon-removal-source-materials/blob/main/Project%20Applications/2022%20Fall/%5BKodama%20Systems%5D%20Frontier%20Carbon%20Removal%20Purchase%20Application.pdf.

105. US EPA, “GHG emission factors hub 2025” (US EPA, 2025); www.epa.gov/system/files/documents/2025-01/ghg-emission-factors-hub-2025.pdf.

106. M. Daniel, T. Gallagher, D. Mitchell, T. McDonald, B. Via, Productivity and cost estimates for incorporating tracked processors into conventional loblolly pine harvesting regimes in the Southeastern United States. *Int. J. For. Eng.* **30**, 155–162 (2019).

107. M. R. Ghaffariyan, Predicting productivity of timber loading operations: A literature review. *Silva Balcanica* **22**, 81–90 (2021).

108. I. Teodorescu, R. Erbaşu, J. M. Branco, D. Tăpuşi, Study in the changes of the moisture content in wood. *IOP Conf. Ser. Earth Environ. Sci.* **664**, 012017 (2021).

109. G. Matthews, *The Carbon Content of Trees* (Forestry Commission, 1993).

110. P. C. Badger, P. Fransham, Use of mobile fast pyrolysis plants to densify biomass and reduce biomass handling costs—A preliminary assessment. *Biomass Bioenergy* **30**, 321–325 (2006).

111. O. Franzese, D. Davidson, “Effect of weight and roadway grade on the fuel economy of class-8 freight trucks” (ORNL/TM-2011/471, Oak Ridge National Laboratory, 2011); https://doi.org/10.2172/1029954.

112. D. Killer, S. Paxton, A. Dawson, Engineering and Legislative Options for Improved Timber Haulage in Scottish Forests: Inclusive Stakeholder Approach. *Transp. Res. Rec.* **1819**, 109–117 (2003).

113. Caterpillar, *Caterpillar Performance Handbook* (Caterpillar, 2016).

114. J. M. van Bemmelen. Die absorption. Das wasser in den kolloïden, besonders in dem gel der kieselsäure *Z. Anorg. Chem.* **13**, 233–356 (1897).

115. W. P. Wadman, S. de Haan, Decomposition of organic matter from 36 soils in a long-term pot experiment. *Plant and Soil* **189**, 289–301 (1997).

116. H. W. Liu, Y. Huang, S. Feng, S. Q. You, Y. Hong, L. D. Shen, Experimental study of methane oxidation efficiency in three configurations of earthen landfill cover through soil column test. *Waste Manag.* **190**, 370–381 (2024).

117. A. A. Hagberg, D. A. Schult, P. J. Swart, "Exploring network structure, dynamics, and function using NetworkX," in *Proceedings of the 7th Python in Science Conference*, G. Varoquaux, T. Vaught, J. Millman, Eds. (SciPy, 2008), pp. 11–15.

118. E. W. Dijkstra, A note on two problems in connexion with graphs. *Numer. Math.* **1**, 269–271 (1959).

119. F. Pan, H.-S. Han, L. R. Johnson, W. J. Elliot, Production and cost of harvesting, processing, and transporting small-diameter (< 5 inches) trees for energy. *For. Prod. J.* **58**, 47–53 (2008).

120. J. Petitmermet, J. Sessions, J. Bailey, R. Zamora-Cristales, Cost and productivity of tethered cut-to-length systems in a dry-forest fuel-reduction treatment: A case study. *For. Sci.* **65**, 581–592 (2019).

121. R. M. Campbell, N. M. Anderson, Comprehensive comparative economic evaluation of woody biomass energy from silvicultural fuel treatments. *J. Environ. Manag.* **250**, 109422 (2019).

122. L. Rodrigues, A. Budai, L. Elsgaard, B. Hardy, S. G. Keel, C. Mondini, C. Plaza, J. Leifeld, The importance of biochar quality and pyrolysis yield for soil carbon sequestration in practice. *Eur. J. Soil Sci.* **74**, e13396 (2023).

123. *M. Elias*, D. L. Sanchez, P. Saksa, J. Hunt, J. Remucal, Market analysis of coupled biochar and carbon credit production from wildfire fuel reduction projects in the westernUSA. *Biofuels Bioprod. Biorefin.* **18**, 1226–1237 (2024).

124. J. R. Trapero, A. Alcazar-Ruiz, F. Dorado, L. Sanchez-Silva, Biochar price forecasting: A novel methodology for enhancing market stability and economic viability. *J. Environ. Manag.* **377**, 124681 (2025).

125. J. Lundgren, B. Vreugdenhil, Y. Ganjkhanlou, R. Baldwin, "Biomass gasification for hydrogen production" (Task 33, IEA Bioenergy, 2025); www.ieabioenergy.com/wp-content/uploads/2025/03/IEA-Bioenergy_T33_Bio-H2_Final_v2.pdf.

126. C. Rodriguez Franco, D. S. Page-Dumroese, D. Pierson, M. Miller, T. Miles, Policy and regulations for mobile biochar production in the United States of America. *Forests* **15**, 192 (2024).

127. S. H. Lamlom, R. A. Savidge, A reassessment of carbon content in wood: Variation within and between 41 North American species. *Biomass Bioenergy* **25**, 381–388 (2003).

128. P. D. Miles, W. B. Smith, "Specific gravity and other properties of wood and bark for 156 tree species found in North America" (Res. Note NRS-38, US Department of Agriculture, Forest Service, Northern Research Station, 2009), pp. 1–35.
